# A ligand-based system for receptor-specific delivery of proteins

**DOI:** 10.1038/s41598-019-55797-1

**Published:** 2019-12-16

**Authors:** Mariano Maffei, Chiara Morelli, Ellie Graham, Stefano Patriarca, Laura Donzelli, Balint Doleschall, Fernanda de Castro Reis, Linda Nocchi, Cora H. Chadick, Luc Reymond, Ivan R. Corrêa, Kai Johnsson, Jamie A. Hackett, Paul A. Heppenstall

**Affiliations:** 1 European Molecular Biology Laboratory (EMBL) Rome, Adriano Buzzati-Traverso Campus, 00015 Monterotondo, Italy; 2 Biomolecular Screening Facility, Ecole Polytechnique Fédérale de Lausanne (EPFL), 1015 Lausanne, Switzerland; 3 National Center of Competence in Research (NCCR) in Chemical Biology, 1015 Lausanne, Switzerland; 4 New England Biolabs, Ipswich, MA 01938 USA; 5 Department of Chemical Biology, Max Plank Institute for Medical Research, 69120 Heidelberg, Germany; 6 Collaboration for joint PhD degree between EMBL and Heidelberg University, Faculty of Biosciences, Heidelberg, Germany

**Keywords:** Biochemistry, Biological techniques, Biotechnology, Cell biology, Chemical biology, Molecular biology

## Abstract

Gene delivery using vector or viral-based methods is often limited by technical and safety barriers. A promising alternative that circumvents these shortcomings is the direct delivery of proteins into cells. Here we introduce a non-viral, ligand-mediated protein delivery system capable of selectively targeting primary skin cells *in-vivo*. Using orthologous self-labelling tags and chemical cross-linkers, we conjugate large proteins to ligands that bind their natural receptors on the surface of keratinocytes. Targeted CRE-mediated recombination was achieved by delivery of ligand cross-linked CRE protein to the skin of transgenic reporter mice, but was absent in mice lacking the ligand’s cell surface receptor. We further show that ligands mediate the intracellular delivery of Cas9 allowing for CRISPR-mediated gene editing in the skin more efficiently than adeno-associated viral gene delivery. Thus, a ligand-based system enables the effective and receptor-specific delivery of large proteins and may be applied to the treatment of skin-related genetic diseases.

## Introduction

Intracellular delivery of biologically active cargoes is of fundamental importance for multiple research and clinical applications. Direct access to the interior of a cell enables, among others, gene editing^1,2^, modulation of gene expression^3^ and *ex-vivo* cell therapies^4^. Realization of these applications is commonly achieved by delivery of exogenous nucleic acids or virus-based methods. Despite their broad use, these techniques often present technical and safety drawbacks such as immunogenicity, risk of permanent integration (genotoxicity) or off-target effects. Moreover, viral vectors have limitations in cargo size narrowing their efficacy. In contrast, protein-based approaches substantially reduce those risks, and indeed, over the last three decades, proteins have emerged as a new class of therapeutic drugs^5–7^.

There are several obstacles for direct protein delivery into cells including cellular internalization and the ability to reach the cytosol of the cell. To overcome these hurdles, proteins can be delivered via physical (e.g. electroporation, microinjection) and biochemical modalities (e.g. pore-forming agents, cell-penetrating peptides)^8^. However, those methodologies are often restricted to *in-vitro* applications or can expose the cell to harsh treatments that are toxic. Most importantly, they lack selectivity, a critical parameter for specific targeting of cells in complex *in-vivo* environments, especially in clinical applications. Thus, efficient intracellular delivery of functional, intact proteins remains a major technological challenge. A valid alternative for instance, is the use of molecular Trojan horse technology where receptor-specific monoclonal antibodies are genetically fused to biologics for their selective delivery across the brain blood barrier^9,10^. Two recent studies have also employed a ligand-mediated approach for targeted delivery of large cargoes *in-vitro*. In a first study Chen and collaborators conjugated the human transferrin protein to a zinc-finger nuclease to perform cell-type specific genome editing^11^. Similarly, an engineered version of Cas9 fused to the asialoglycoprotein ligand was selectively delivered in liver cells through a receptor-mediated internalization mechanism^12^. Both works highlighted the many advantages of using a ligand-based platform including good selectivity, low cell-toxicity and precise temporal control. However, the employment of these systems *in-vivo* represents the next challenge.

In this study, we aimed to develop a receptor-specific targeting tool using the skin as a model for *ex-vivo* and *in-vivo* delivery. Skin is the largest organ of the body and plays both a protective and sensory role in interacting with the external environment. Keratinocytes are the major cell type of this organ, and these cells constantly cycle to maintain a functional barrier that protects against invading pathogens such as virus or bacteria. Despite the accessibility of the skin, keratinocytes are not amenable to most of the standard delivery methodologies, and they have proven to be extremely difficult to target with external molecules^13^. This in turn limits the development of strategies for effective therapies of skin-related diseases. In this light, a ligand-based system could represent a key technology to gain access to keratinocytes, enabling novel therapeutic applications in the skin.

We previously described a protein based tool for the delivery of a small molecule photosensitizer to the skin, along with a light-mediated control of itch and inflammatory skin disease^14^. This approach was based upon a SNAP-tagged engineered version of the cytokine interleukin-31 (IL-31_K138A_SNAP), that binds to its receptors (IL31RA and OSMR) on keratinocytes, but does not provoke cellular signaling. Here, we have asked whether an analogous approach might also be used to deliver large, biologically active proteins to keratinocytes. We found that IL-31_K138A_SNAP is translocated to the nucleus of primary murine keratinocytes upon internalization. We further identified a second non-signaling ligand (Nerve Growth Factor R121W; NGF_R121W_SNAP)^15^ that also binds to keratinocytes and is translocated to the nucleus. Together, these observations suggested that conjugation of cargoes to IL-31_K138A_SNAP or NGF_R121W_SNAP might allow for their intracellular uptake in primary keratinocytes. To test this, we generated recombinant CLIP-tagged CRE recombinase and Cas9 nuclease, to enable their chemical crosslinking to SNAP-tagged ligands using bifunctional benzylcytosine (BC) and benzylguanine (BG) substrates^16^. We demonstrate that cross-linked complexes are selectively delivered into primary keratinocytes both *in-vitro* and *in-vivo* and can achieve cell-type specific gene editing including homology-directed repair *in-vivo*.

## Results

### Nuclear translocation of IL-31_K138A_SNAP and NGF_R121W_SNAP in keratinocytes

**Figure 1:**
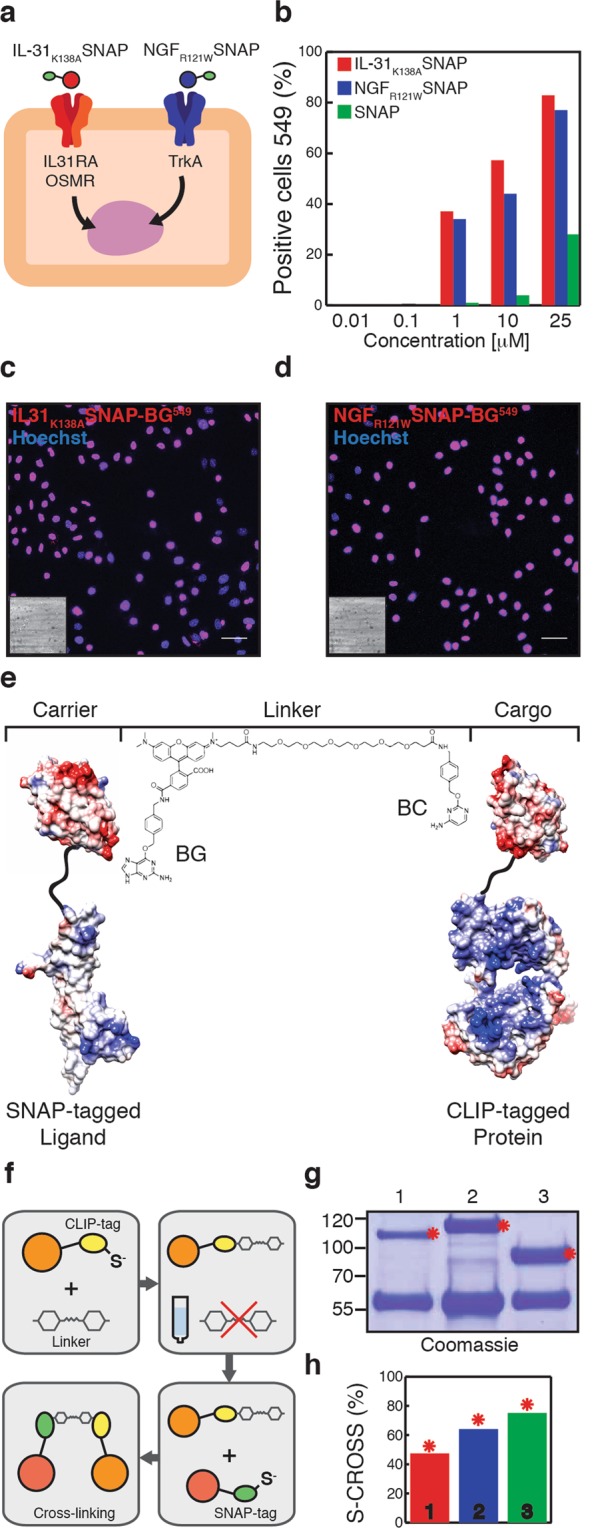
Binding of SNAP-tagged ligands to keratinocytes and selective cross-linking to CLIP-tagged enzymes. (**a**) Schematic representation of one keratinocyte expressing the receptors of interest and the ligands used. (**b**) Quantification of labelled IL-31_K138A_SNAP-BG^549^ (**c**), NGF_R121W_SNAP-BG^549^ (**d**) and SNAP-BG^549^ (green bars) binding to primary keratinocytes. Nuclear localization was observed after 2 hours treatment. The nuclei were stained with Hoechst. Scale bars, 20 μm. The insets represent corresponding brightfield images. (**e**) 3D structures showing selective cross-linking of SNAP-tagged ligands (NGF-SNAP) and CLIP-tagged proteins (CLIP-Cre) through a BG-TMR-PEG-BC linker (PDB ID codes: 1BET, 1KBU, 3KZY). (**f**) Schematic representation of S-CROSS optimized chemical reaction. (**g**) Representative Coomassie gel showing cross-linking complexes (red asterisks). First lane (#1) is IL-31^SNAP::CLIP^CRE, second lane (#2) is NGF^SNAP::CLIP^CRE and third lane (#3) is SNAP::CLIP-CRE. (**h**) Quantification of cross-linking from Coomassie gel (**g**).

A critical step for the delivery of functional proteins is the ability to gain access to the interior of a cell upon uptake. While performing live imaging experiments to characterize the binding and internalization of BG-Surface^549^ (BG^549^) labelled IL-31_K138A_SNAP to keratinocytes (Fig. S1A), we observed a robust translocation of the ligand to the nucleus of cells over the course of a 2 hour incubation (Fig. 1a–c). Similarly, we investigated whether a second ligand (NGF_R121W_SNAP)^15^ was able to bind to keratinocytes through recognition of its natural receptors which are also highly expressed in this type of cells^17^. BG-Surface^549^ labelled NGF_R121W_SNAP (Fig. S1B) was applied to primary mouse keratinocytes at increasing concentrations. As expected and similar to previous reported studies^18^, we also detected a strong fluorescence in the cell nucleus upon prolonged incubation (2 hours) with the NGF-probe (Fig. 1a,b,d). In contrast, minimal cellular internalization was observed when cells were treated with labelled SNAP-tag alone (in the absence of ligand) or with BG-Surface^549^ (Figs. 1b and S1C,D). Cellular internalization of SNAP-tagged ligands of interest was further confirmed by Western Blot analysis (Fig. S1G). Thus, IL-31_K138A_SNAP and NGF_R121W_SNAP ligands bind to primary keratinocytes and are translocated to the nucleus, enabling intracellular access.

### Chemical cross-linking of SNAP-ligands to CLIP-Cre

We reasoned that the rapid nuclear translocation of SNAP-ligands might be exploited to allow for targeted delivery of biologically active protein cargoes in primary keratinocytes. To this end, we first attempted to generate fusion proteins of ligand and CRE recombinase but were unable to recover active proteins at sufficient yields. We therefore asked whether selective cross-linking (S-CROSS)^16^ could be employed to bind molecules of interest. S-CROSS is based on self-labelling tags (SNAP and CLIP), which covalently bind synthetic probes and has been previously used to detect protein-protein interactions in living cells^16,19^. Recombinant CRE recombinase fused to a N-terminal CLIP-tag (CLIP-Cre) was produced in *E*. *Coli* and CLIP activity was confirmed by selective labelling with a BC-derivative fluorophore (BC^488^) (Fig. S1E). S-CROSS was next assessed *in-vitro* by mixing molar equivalents of CLIP-Cre with SNAP-ligands together with cross-linker molecules carrying both BG and BC moieties on their ends, as schematically shown in Fig. 1e. We screened several cross-linker candidates in order to identify the synthetic probe that allowed the highest yield of S-CROSS (Table S1 and Fig. S1F). We determined that long linkers (>25 Å; linker #2, #3, #5, #6; Table S1) were more effective for S-CROSS, most likely because they reduce steric hindrance thus allowing the reactive groups (BG and BC) to have better access to the SNAP and CLIP tags. In particular, linker #5^20^ (Table S1) was found to display the highest rate of S-CROSS. Finally, optimization of the cross-linking process was achieved through a two-step reaction (Fig. 1f): CLIP-tagged cargo was firstly saturated with the cross-linker (linker #5) and, after elimination of the unbound compound, SNAP-ligands were added to the reaction mix. Up to 60% cross-linking was obtained with no excess of free SNAP-ligand present in the final product (Fig. 1g,h).

### Ligand-mediated selective delivery of CLIP-Cre *in-vitro*

**Figure 2:**
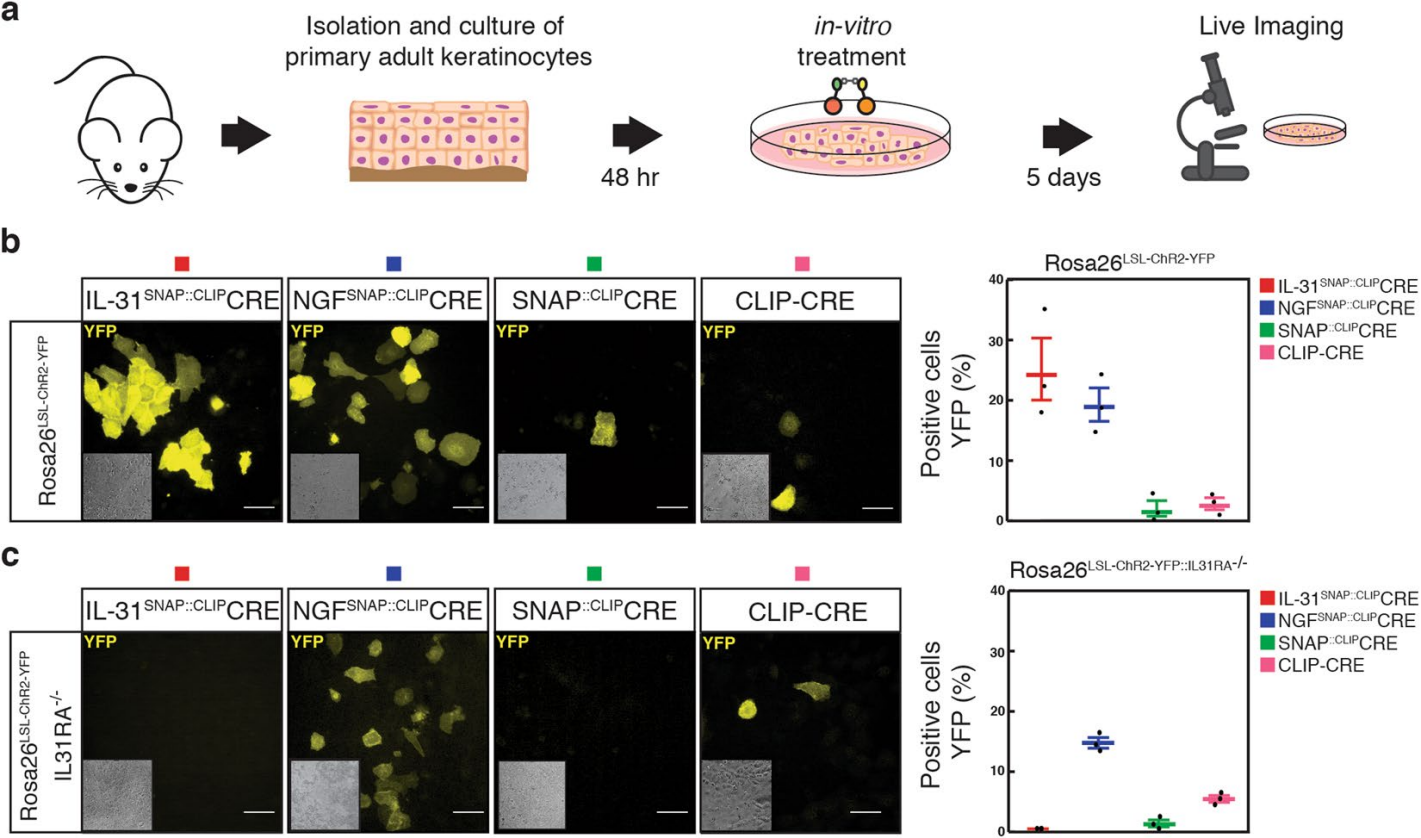
Ligand-mediated delivery of cross-linked CLIP-Cre. (**a**) Schematic of *in-vitro* keratinocytes treatment with cross-linked complexes. (**b**) Images and quantification of YFP positive primary keratinocytes (% of cells) from Rosa26^LSL-ChR2-YFP^ mice 5 days after treatment with 2 μM of cross-linked complexes or CLIP-Cre alone. (**c**) Representative images and quantification of YFP positive primary keratinocytes from double transgenic Rosa26^LSL-ChR2-YFP::IL31RA−/−^ mice 5 days after treatment with 2 μM of cross-linked complexes or CLIP-Cre alone. Scale bars, 20 μm. The insets represent corresponding brightfield images. The horizontal lines mark the geometric mean and the error bars mark the standard error. Representative data from *n* = 3 independent experiments.

To assess whether targeted delivery of cross-linked CLIP-Cre to ligands of interest was functional, we applied S-CROSS complexes to primary adult murine keratinocytes cultured from Rosa26^LSL-ChR2-YFP^ reporter mice, in which yellow fluorescent protein (YFP) expression is induced upon CRE-mediated recombination. CLIP-Cre, cross-linked *in-vitro* to either IL-31_K138A_SNAP (IL-31^SNAP::CLIP^CRE; linker #5, Table S1) or NGF_R121W_SNAP (NGF^SNAP::CLIP^CRE; linker #5, Table S1) was applied to keratinocytes and after 5 days YFP expression was assessed (Fig. 2a). Upon a single *in-vitro* treatment we observed 26.5% ± 4.9 expression of reporter YFP for IL-31^SNAP::CLIP^CRE complex and 20.0% ± 2.6 when cells were treated with NGF^SNAP::CLIP^CRE S-CROSS (Fig. 2b). Of note, the percentage of targeted cells was similar to the number of keratinocytes labelled with free SNAP-tagged ligands (Fig. 1c; 1 μM condition). Importantly, negligible YFP expression (0–3%) was detected upon incubation with a cross-linked binary complex lacking ligands of interest (SNAP::CLIP-CRE; linker #5, Table S1) or when CLIP-Cre alone was applied to keratinocytes (Fig. 2b) suggesting that S-CROSS internalization is primarily driven by ligands. To confirm the selectivity of the system, we used a Rosa^26LSL-ChR2-YFP^ reporter mouse model lacking the Interleukin 31 receptor alpha subunit (IL31RA−/−)^14^. Rosa26^LSL-ChR2-YFP^ mice were crossed with IL31RA−/− mice and primary keratinocytes were cultured. S-CROSS (IL-31^SNAP::CLIP^CRE or NGF^SNAP::CLIP^CRE; linker #5, Table S1) was applied to keratinocytes and YFP expression was assessed. Strikingly, no YFP activation was detected on cells treated with the cross-linking complex carrying the IL-31 ligand, while NGF S-CROSS displayed a similar YFP activation as for wild-type Rosa26^LSL-ChR2-YFP^ keratinocytes (Fig. 2c). Thus, SNAP-tagged ligands mediate selective intracellular delivery of CLIP-Cre *in-vitro*.

### Ligand-mediated selective delivery of CLIP-Cre *in-vivo*

**Figure 3:**
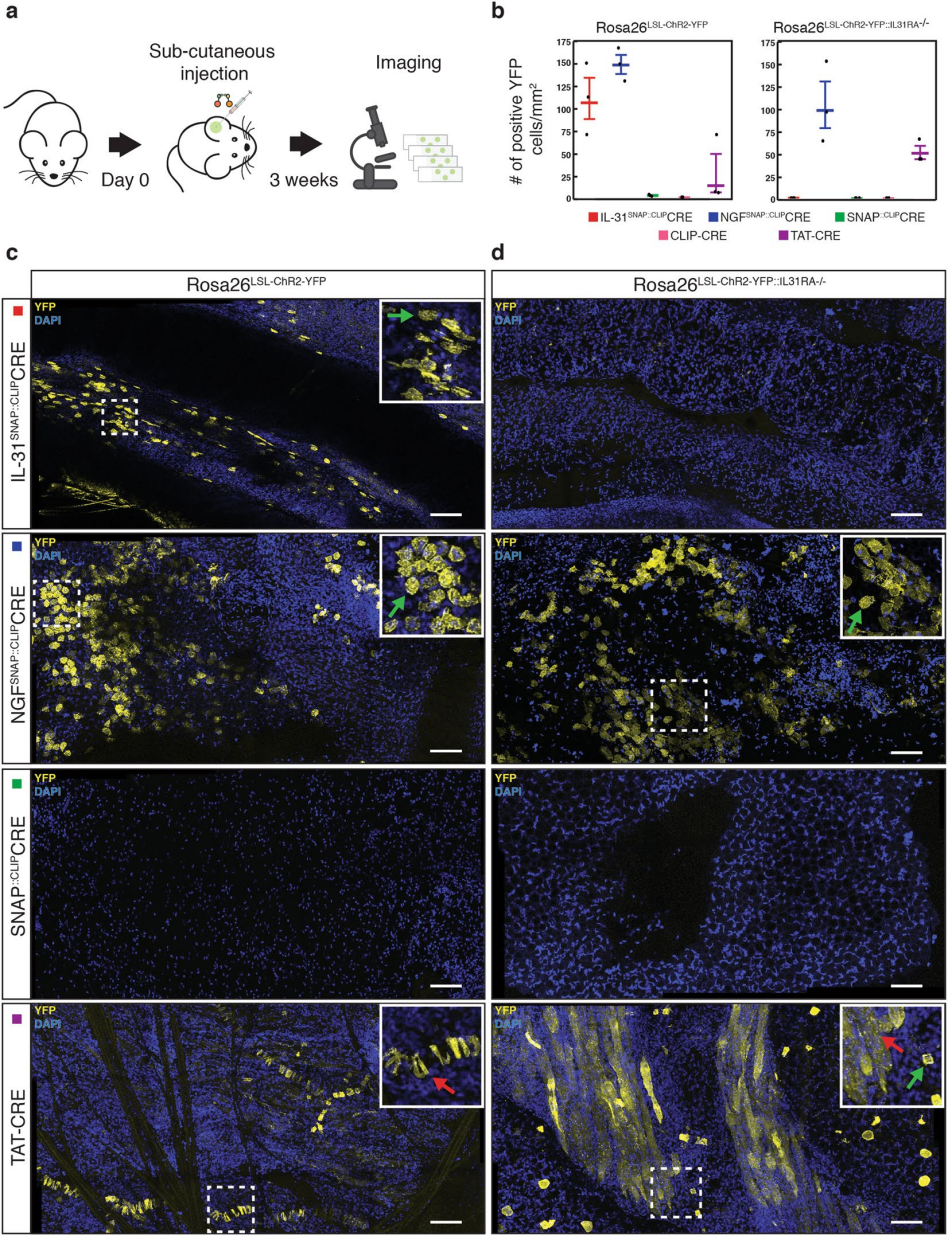
Selective delivery of cross-linked CLIP-Cre *in-vivo*. (**a**) Schematic of *in-vivo* treatment with cross-linked complexes. (**b**) Quantification (number of cells per mm^2^) of YFP positive keratinocytes from Rosa26^LSL-ChR2-YFP^ mice (**c**) and from double transgenic Rosa26^LSL-ChR2-YFP::IL31RA−/−^ mice (**d**) 3 weeks after subcutaneous injection with 5 μM (0.85 mg/kg) of cross-linked complexes or TAT-Cre. The nuclei were stained with DAPI. Scale bars, 40 μm. The insets show the zoom of representative areas. Green arrows indicate YFP+ keratinocytes. Red arrows indicate non-selective YFP expression. The horizontal lines mark the geometric mean and the error bars mark the standard error. Data from *n* = 3 independent experiments.

We next investigated whether IL-31_K138A_SNAP or NGF_R121W_SNAP ligands can drive intracellular delivery of CLIP-Cre *in-vivo* in mice. S-CROSS reactions (IL-31^SNAP::CLIP^CRE or NGF^SNAP::CLIP^CRE; linker #5, Table S1) were injected subcutaneously into the ear of Rosa26^LSL-ChR2-YFP^ reporter mice and after 3 weeks, YFP expression was assessed by confocal microscopy on whole mount samples (Fig. 3a). Upon a single treatment, broad YFP expression was detected in keratinocytes of both mice injected with IL-31^SNAP::CLIP^CRE and NGF^SNAP::CLIP^CRE (Fig. 3b left panel and c). No YFP signal was observed after injection with SNAP::CLIP-CRE complex or CLIP-Cre alone (Fig. 3b left panel and C and Fig. S2A). Moreover, subcutaneous injection of a recombinant cell-permeant peptide fusion CRE-recombinase protein^21,22^ (TAT-Cre) led to a non-cell specific YFP expression and displayed lower efficiency compared with ligand-driven delivery (Fig. 3b left panel and c). We further validated cell-type specific delivery by injecting S-CROSS into the ear of double transgenic IL31RA knockout/reporter mice (Rosa26^LSL-ChR2-YFP::IL31RA−/−^). No YFP signal was observed upon injection with IL-31^SNAP::CLIP^CRE complex whereas reporter activation was maintained for NGF-mediated delivery of CLIP-Cre (Fig. 3B right panel and d). These results demonstrate that ligand-mediated delivery is functional and selective also *in-vivo*.

### Chemical cross-linking of SNAP-ligands to CLIP-Cas9

**Figure 4:**
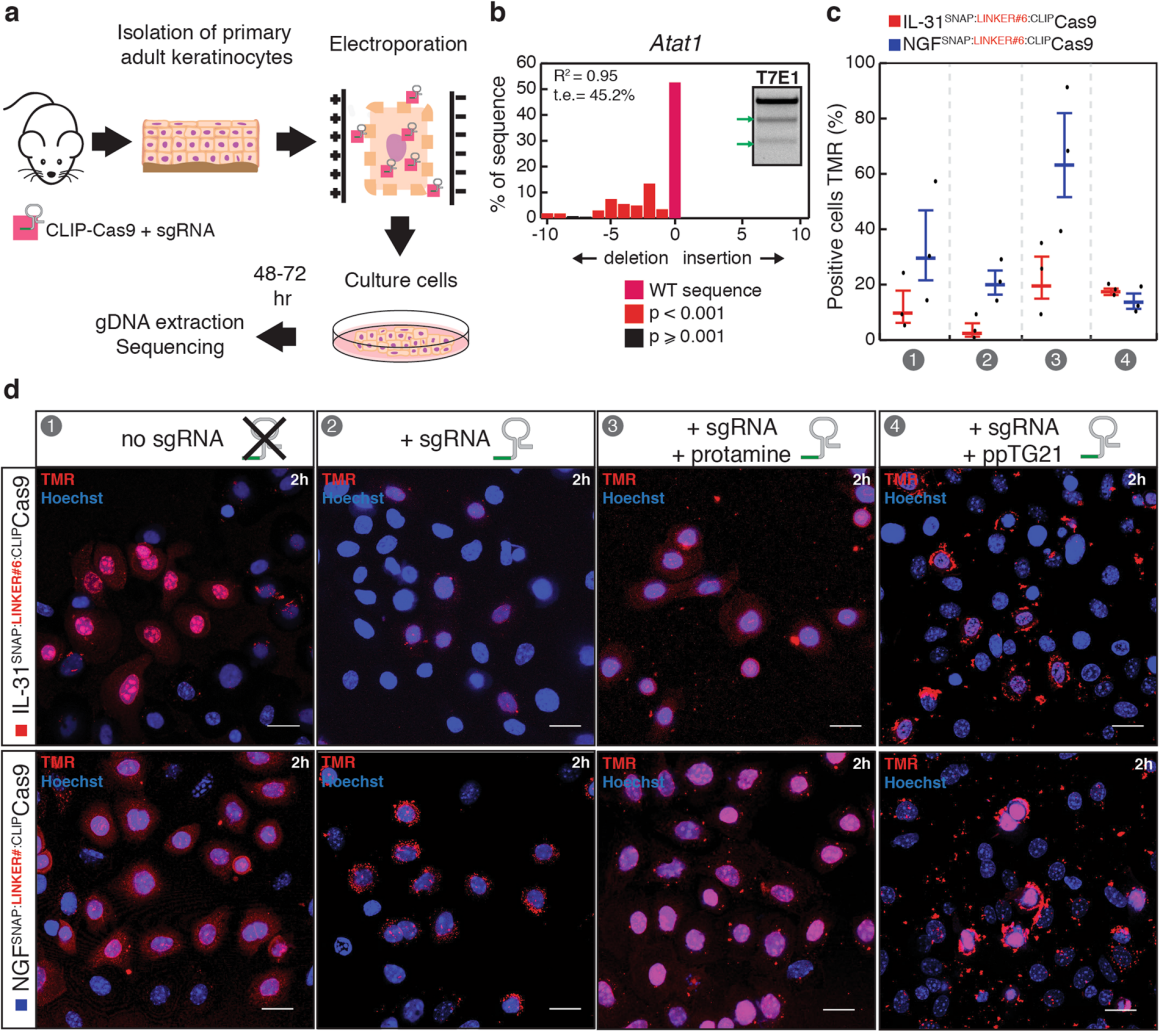
CLIP-Cas9 activity and internalization in keratinocytes. (**a**) Schematic of CLIP-Cas9::sgRNA electroporation strategy. (**b**) Indel spectrum determined by TIDE of primary keratinocytes electroporated with CLIP-Cas9::sgRNA targeting the Atat1 gene. The inset show T7 endonuclease 1 assay performed on genomic DNA from electroporated keratinocytes. t.e. = total efficiency. (**c**) Quantification (% cells) and representative images (**d**) of TMR positive cells upon 2 hours treatment with 2 μM of ligand cross-linked Cas9 (#1 no sgRNA; #2 with sgRNA; #3 with sgRNA+ protamine; #4 with sgRNA + ppTG21). Nuclei were stained with Hoechst. Scale bars, 20 μm. The horizontal lines mark the geometric mean and the error bars mark the standard error.

We next asked whether SNAP-ligand cross-linking can also be used to deliver Cas9 nuclease intracellularly. Similar to CLIP-Cre, a recombinant version of Cas9 nuclease fused to an N-terminal CLIP-tag (CLIP-Cas9) was produced in *E*. *Coli*. Efficient labelling with a BC derivative fluorophore (BC^TMR^) was observed indicating that the CLIP-tag was active (Fig. S3A). The functionality of CLIP-Cas9 was then assessed at the *in-vitro* level and in-cell, by monitoring nuclease activity at the mouse Atat1 locus. In an *in-vitro* digestion assay (IDA)^23^ using Atat1 PCR product and chemically synthesized dual RNAs (Atat1 crRNA and trRNA) we observed efficient cleavage of the PCR product (Fig. S3E). We tested recombinant CLIP-Cas9 nuclease activity in-cell by direct electroporation of preassembled CLIP-Cas9::sgRNA (Atat1 crRNA and trRNA) ribonucleoprotein (RNP) complexes in primary keratinocytes isolated from C57BL/6J WT adult mice (Fig. 4a). Gene editing was observed by tracking of indels by decomposition analysis (TIDE)^24^ of the PCR amplicons from the Atat1 genomic locus and confirmed by T7 endonuclease 1 (T7E1) assay (Figs. 4b and S3B). Of note, CLIP-Cas9 gene editing efficiency (45.2%) was comparable to native Cas9 activity (60.2%) when the latter was electroporated together with sgRNA (Cas9::sgRNA) in keratinocytes (Fig. S3C). We further assessed selective cross-linking of ligands with recombinant CLIP-Cas9. Similar to CLIP-Cre S-CROSS, we obtained up to 35% of cross-linked CLIP-Cas9 to IL-31_K138A_SNAP (IL-31^SNAP::CLIP^Cas9, linker #5, Table S1) and about 55% to NGF_R121W_SNAP (NGF^SNAP::CLIP^Cas9, linker #5, Table S1) (Fig. S3D). Thus, validation assays showed that CLIP-Cas9 was functional and efficiently conjugated to ligands of interest.

### Internalization of ligand cross-linked CLIP-Cas9 complexes

To determine whether Cas9 S-CROSS could be internalized in keratinocytes, CLIP-Cas9 was cross-linked *in-vitro* to either IL-31_K138A_SNAP (IL-31^SNAP::CLIP^Cas9) or NGF_R121W_SNAP (NGF^SNAP::CLIP^Cas9) using a linker carrying a TMR fluorophore (linker #6^25^; Table S1) to monitor internalization (Fig. S4A). IL-31^SNAP::CLIP^Cas9 or NGF^SNAP::CLIP^Cas9 (linker #6) were applied to cultured murine WT primary keratinocytes, and after 2 hours of incubation, S-CROSS uptake was assessed using live cell imaging. Red fluorescence was observed inside the cells, mostly localized in the nuclei suggesting that the protein complex was internalized (Fig. 4c,d, condition #1). We next investigated whether ribonucleoprotein complexes (IL-31^SNAP::CLIP^Cas9 or NGF^SNAP::CLIP^Cas9 bound to sgRNA targeting Atat1 gene) were taken up by primary keratinocytes. Ligand cross-linked CLIP-Cas9 complexes were incubated *in-vitro* with sgRNA (IL-31^SNAP::CLIP^Cas9::sgRNA or NGF^SNAP::CLIP^Cas9::sgRNA), applied to cultured cells, and internalization was assessed after 2 hours. Compared to ligand-Cas9 complexes alone, an apparent reduction of the fluorescent intracellular signal was observed for both complexes upon addition of sgRNA (Fig. 4c,d, condition #2). Decreased S-CROSS internalization in the presence of sgRNA was further confirmed by Western blotting analysis (Fig. S4B). As expected, and similar to a previous study performed in primary cells^12^, no gene editing at the Atat1 locus was detected in keratinocytes using ligand-Cas9 RNP complexes (Fig. S4F). Taken together, these data indicate that ligands can mediate cell-specific delivery of Cas9 protein, however the presence of sgRNA interferes with RNP internalization mechanisms. To address this issue, we selected two candidate peptides that have previously been described to promote internalization, protamine and ppTG21, and investigated whether RNP delivery was improved in their presence. Protamine is a small positively charged peptide which binds with high affinity to nucleic acids and has been previously employed for small and long RNA delivery^26,27^ (Fig. S4C). ppTG21 is an endosomolytic peptide demonstrated to enhance endosomal escape of Cas9 RNP complexes whilst maintaining selectivity^12^. Preassembled IL-31^SNAP::CLIP^Cas9::sgRNA or NGF^SNAP::CLIP^Cas9::sgRNA were incubated *in-vitro* with an excess of protamine and then applied to cultured cells. After 2 hours of treatment, an increase of fluorescent signal was observed in the nuclei of live cells suggesting that co-incubation with protamine can favour cellular internalization of RNP complexes (Fig. 4c,d, condition #3). We further examined S-CROSS::sgRNA complex internalization in the presence of ppTG21. Preassembled IL-31^SNAP::CLIP^Cas9::sgRNA or NGF^SNAP::CLIP^Cas9::sgRNA were co-incubated *in-vitro* with 30 molar equivalent of ppTG21 peptide, applied to cells and imaged 2 hours later. In contrast to protamine, ppTG21 did not improve RNP internalization (Fig. 4c,d, condition #4). Negligible internalization was also observed in control conditions when TMR-labelled CLIP-Cas9 (CLIP-Cas9 + linker #6) was applied to cells in the absence of ligand (with or without sgRNA, Protamine, or ppTG21 (Fig. S4D,E, conditions #1–4). Finally, we assessed in-cell gene editing at the Atat1 locus using ligand-Cas9 complexes delivered under the conditions described above. Unexpectedly, while we found that protamine facilitates internalization, sequencing data indicated that RNP complexes were no longer functional (Fig. S4F). We thus hypothesized that protamine sequesters or blocks the interaction of sgRNA with Cas9 or its target.

### In-cell gene editing using ligand-mediated delivery of CLIP-Cas9

**Figure 5:**
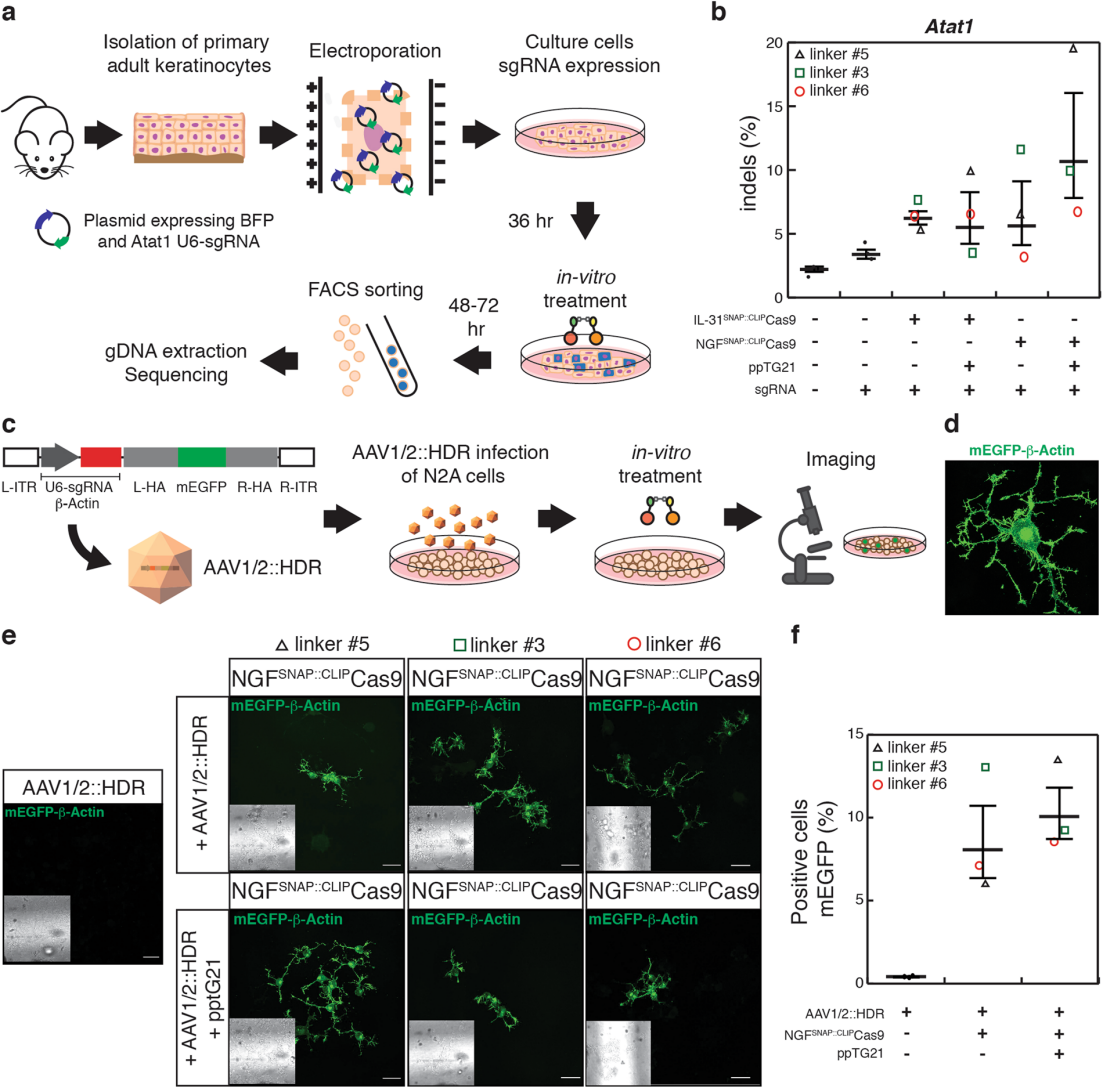
Ligand-mediated delivery of cross-linked CLIP-Cas9 in cultured cells. (**a**) Schematic of plasmid electroporation strategy in primary keratinocytes. (**b**) % of indels detected from DNA sequencing of BFP-sorted keratinocytes expressing Atat1 U6-sgRNA and treated with: cross-linked complex IL-31^SNAP::CLIP^Cas9 (3rd column from left), IL-31^SNAP::CLIP^Cas9 + ppTG21 (4^th^ column from left), NGF^SNAP::CLIP^Cas9 (5^th^ column from left) and NGF^SNAP::CLIP^Cas9 + ppTG21 peptide (6^th^ column from left). Untreated keratinocytes and untreated BFP-sorted keratinocytes are shown in column 1 and 2, respectively. (**c**) Graphical representation of AAV1/2::HDR and N2a cells experimental strategy. (**d**) Zoom of mEGFP-ß-Actin N2a positive cell. (**e**) Confocal images of AAV1/2::HDR transduced N2a WT cells overexpressing NGF receptors treated with NGF^SNAP::CLIP^Cas9 alone (upper frames) or in presence of ppTG21 peptide (lower frames). First images on the left show control N2a cells infected only with AAV1/2::HDR. Scale bars, 40 μm. The insets represent corresponding brightfield images. (**f**) Quantification (% cells) of mEGFP positive cells from (**e**). The horizontal black lines mark the geometric mean and the error bars mark the standard error. Black triangles: cross-linking complexes carrying linker #5 (Table S1); green squares: cross-linking complexes carrying linker #3 (Table S1); red circles: cross-linking complexes carrying linker #6 (Table S1).

Based on the above observation, we reasoned that the sgRNA may have to be provided independently to achieve in-cell gene editing upon ligand mediated delivery of Cas9. To test this hypothesis, we electroporated a plasmid encoding Blue Fluorescent Protein (BFP) as a transfection marker, and a U6 promoter driven sgRNA targeting the Atat1 locus (U6-sgRNA) into primary keratinocytes. After 36 hours, and upon expression of BFP in 12.2% ± 0.4 of cells (Fig. S5A,B), ligand conjugated CLIP-Cas9 was applied to keratinocytes. We tested both IL-31_K138A_SNAP and NGF_R121W_SNAP mediated delivery, three different cross-linkers (linker #3, #5 and #6, Table S1), and the endosomolytic peptide ppTG21. 48–72 hours after treatment, BFP positive keratinocytes were sorted by flow cytometry and genomic DNA was extracted to assess editing at the Atat1 locus (Fig. 5a). Gene editing was observed with comparable efficiency for both ligands at significantly greater levels than control conditions (6.2% ± 0.5 indels for IL-31^SNAP::CLIP^Cas9; 6.6% ± 2.4 indels for NGF^SNAP::CLIP^Cas9; 1.9% ± 0.2 indels for control conditions) (Figs. 5b and S5E). We also observed enhanced internalization of ligand cross-linked Cas9 (without sgRNA) in the presence of ppTG21 (Fig. S5C,D), and this was also evident as a substantial improvement in gene editing frequency for linker #5 (19.8% indels for NGF^SNAP::CLIP^Cas9, Figs. 5b and S5E). Thus, supplying sgRNA via a separate route to the ligand-delivered Cas9, allows for gene editing in primary keratinocytes.

### Ligand-mediated delivery of CLIP-Cas9 allows for homology directed repair

As a further readout for gene editing efficiency, in particular for *in-vivo* experiments, we next investigated whether ligand-mediated delivery of CLIP-Cas9 could also be employed for genomic knock-in of a fluorescent reporter through homology-directed repair (HDR)^28^. To achieve this, we used a previously described strategy employing an adeno-associated virus (AAV) containing a U6-sgRNA targeting the mouse ß-Actin gene and a donor template encoding for the monomeric Green Fluorescent Protein (mEGFP) with homology arms for targeted insertion at the N-terminus of ß-Actin^29^ (Fig. 5c). We assessed the approach in murine neuroblastoma Neuro-2a (N2a) cells^30^ overexpressing NGF receptors and shown to be bound by NGF_R121W_SNAP (Fig. S6A). Cells were infected with an AAV serotype 1/2 vector containing ß-Actin sgRNA and donor template (AAV1/2::HDR), and 8–24 hours later, treated with NGF cross-linked CLIP-Cas9 (NGF^SNAP::CLIP^Cas9) (Fig. 5c). Again, gene-editing efficiency was assessed for three different cross-linkers (linker #3, #5 and #6, Table S1) in the presence or absence of the ppTG21 peptide. Remarkably, at 72–96 hours post-treatment we observed mEGFP expression localized to the cytoskeleton in cells (Fig. 5d,e). This was present in 8.9% ± 2.1 of cells (Fig. 5f). Cross-linked complex using linker #3 (Table S1) displayed the highest efficiency (~15%) while linker #5 and #6 were shown to be less effective (~5–7%) (Fig. 5f). Intriguingly, and in agreement with sequencing results (Fig. 5b), co-incubation with ppTG21 led to an increase in HDR efficiency for linker #5 but not for other linkers (Fig. 5f). Importantly, no mEGFP signal was detected in cells infected only with AAV1/2::HDR (0.1% ± 0.1) or treated with CLIP-Cas9 alone (Figs. 5e,f and S6B).

**Figure 6:**
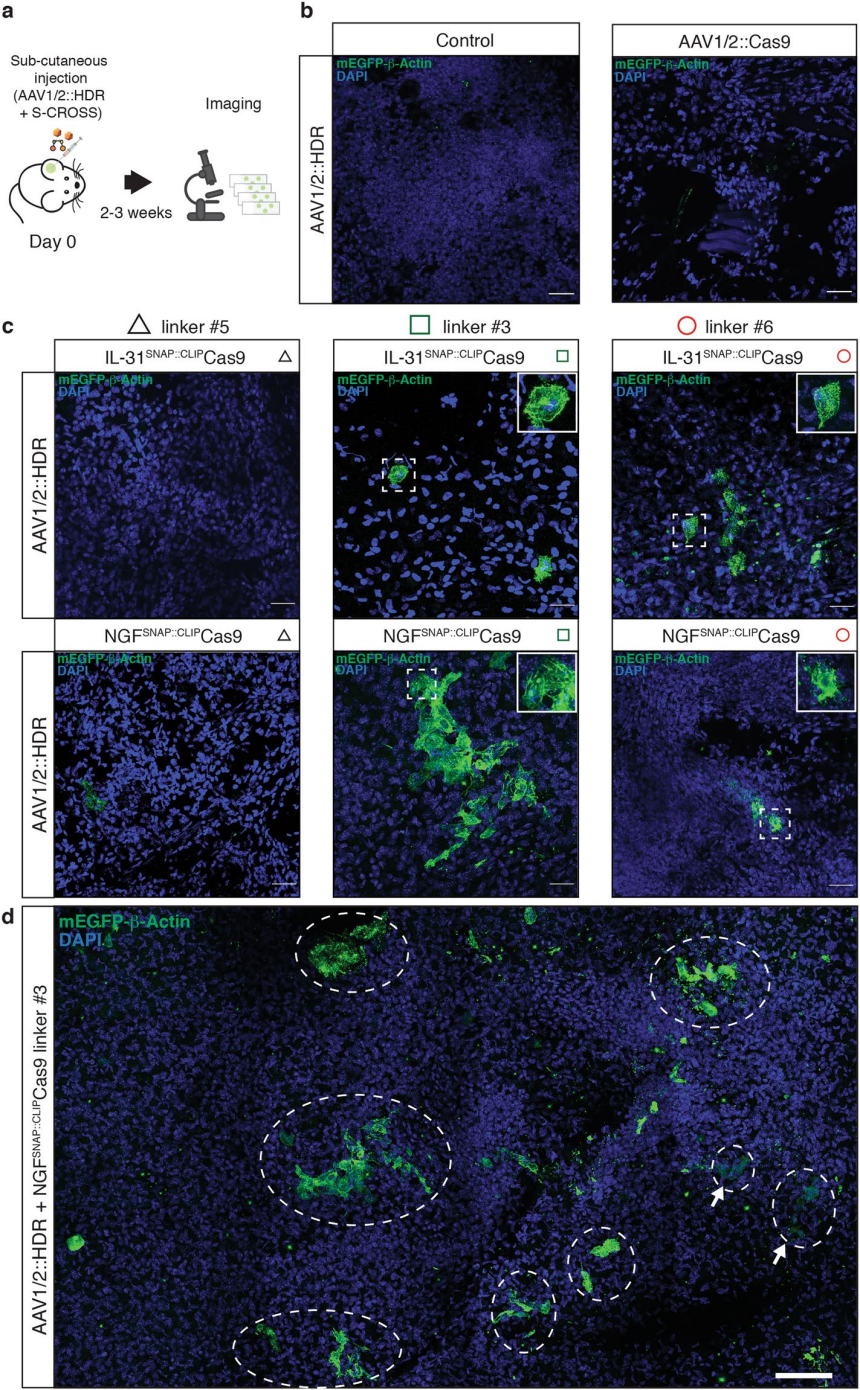
Ligand-mediated delivery of cross-linked CLIP-Cas9 *in-vivo*. (**a**) Schematic of subcutaneous injection of cross-linked CLIP-Cas9 and AAV1/2::HDR. (**b**) Confocal images showing control samples injected only with AAV1/2::HDR (left frame) or with dual AAV system AAV1/2::HDR + AAV1/2::Cas9 (right frame) (**c**) Confocal images of mEGFP positive keratinocytes 2–3 weeks after subcutaneous injection into the mouse ear, with AAV1/2::HDR and CLIP-Cas9 cross-linked to IL-31 (top) or NGF (bottom). The nuclei were stained with DAPI. Scale bars, 40 μm. The insets show enlarged representative areas with mEGFP-ß-Actin positive cells. Black triangles: cross-linking complexes carrying linker #5 (Table S1); green squares: cross-linking complexes carrying linker #3 (Table S1); red circles: cross-linking complexes carrying linker #6 (Table S1). (**d**) Mosaic confocal image (1.07 mm × 1.43 mm) of mEGFP-ß-Actin positive cells from WT mice 2–3 weeks after subcutaneous injection with AAV1/2::HDR and NGF^SNAP::CLIP^Cas9 (linker #3). The nuclei were stained with DAPI. White dashed circles mark mEGFP positive targeted cell clusters. White arrows indicate mEGFP positive cells starting to express mEGFP. Scale bar, 100 μm.

We next investigated whether ligand-mediated delivery of CLIP-Cas9 allows for HDR *in-vivo* in the skin. IL-31^SNAP::CLIP^Cas9 or NGF^SNAP::CLIP^Cas9 (linker #5, #3, #6) were injected subcutaneously into the ear of C57BL/6J WT mice together with the AAV1/2::HDR vector (Fig. 6a). 2–3 weeks post-treatment, we evaluated mEGFP-ß-Actin expression by confocal microscopy on whole mount samples (Fig. 6a). We observed clusters of mEGFP positive keratinocytes around the injection site (Fig. 6c,d), with linker #3 showing the highest efficiency, especially for NGF-mediated delivery (Fig. 6c, green square). HDR was also observed, at lower frequency, in samples injected with cross-linked complexes carrying linker #6 (Fig. 6c, red circle), while no HDR event was detected for S-CROSS linker #5 (Fig. 6c, black triangle). Finally, we benchmarked the ligand-based system against viral delivery of Cas9. AAV1/2::HDR and a second AAV carrying the Cas9 sequence (AAV1/2::Cas9)^29^ were subcutaneously injected into the ear of WT mice, and skin samples were analyzed by confocal microscopy 2–3 weeks later. We detected no mEGFP fluorescence in any skin sample after viral delivery of Cas9 (Fig. 6b), indicating that delivery of Cas9 protein through ligand conjugation may offer a more efficient means of targeting the skin.

## Discussion

Here we present a method for non-viral receptor-dependent delivery of large cargoes chemically conjugated to protein ligands. Based on previous evidence^14^, we have demonstrated that a non-signalling ligand (IL-31_K138A_SNAP) is translocated to the nucleus of primary keratinocytes upon recognition of its natural heterodimeric receptor complex (IL31-RA/OSMR). We have further identified and characterized the ability of a second non-signalling ligand (NGF_R121W_SNAP)^15^ to target keratinocytes. Upon internalization, NGF_R121W_SNAP was also shown to be translocated to the nucleus of the targeted cells. NGF internalization and its nuclear transport through a receptor-mediated endocytosis mechanism was previously reported to occur in neurons^18,31,32^. In addition to providing access to receptor-specific expressing cells, the engineered ligands we used here do not provoke pain or scratching behaviour, thus representing a valuable delivery tool for both *in-vitro* and *in-vivo* applications.

We exploited these ligands to deliver two different cargo proteins, CRE recombinase and Cas9 nuclease into cells. We covalently cross-linked molecules of interest using SNAP and CLIP-tag fusions, together with long bio-orthogonal linkers. The main advantage of this approach is its versatility, allowing the mixing of combinations of “protein building blocks” using a two-step simple chemical reaction and avoiding issues related to the production of large fusion proteins. We have identified three different linkers (linker #3, #5, #6) shown to minimize steric hindrance effects between cargoes and ligands, thus allowing for high yields of cross-linking complexes. In addition to this, selected probes contain functional moieties which enable imaging (TMR, linker #6) and potentially promote the uptake and the endosomal escape of the protein complexes (biotin, linker #3 and #5; TMR, linker #6)^33,34^.

Both IL-31 and NGF ligands displayed efficient delivery of biologics into studied cells which was lost in the absence of receptors, thus demonstrating that the system is receptor-specific. Importantly, they enabled access to keratinocytes, which constitute the major cellular component of the skin and the first barrier from the external environment. Keratinocytes have been shown to be highly resistant to viral infection or to standard established delivery methods (e.g. lipid-based reagents), making them a challenging target. To that end, our system could be a viable tool to target the skin. However, since other cell types in the skin are known to express IL-31 and NGF receptors^17,35–37^, in future studies it will be important to investigate the delivery of cargoes to these cells together with the pharmacodynamics of the system in the skin.

Many keratinocyte associated genetic disorders have been described^38–40^ with no effective therapies available on the market. In this light, the technology we describe here could represent a promising platform for treating those diseases. Furthermore, the direct delivery of Cas9 as a protein has the advantage of minimizing risks of potential integration into genomic DNA and prolonged exposure to the nuclease, that are associated with other delivery methods (viral vectors or plasmid-based technologies), making it a safer alternative. Future improvements will focus on increasing delivery efficiency and on expanding the system to other types of cargoes (for example siRNA and therapeutic proteins). We also expect our strategy could be expanded to distinct biologics (ligands, antibodies or small molecules) that bind to specific cell-surface receptors, thus allowing for selective targeting of many other cellular populations and offering new options to current methodologies.

## Methods

### Animals

Wild type C57BL/6J, Rosa26^LSL-ChR2-YFP^ and Rosa26^LSL-ChR2-YFP::IL31RA−/−^ mice were used at age of 7–15 weeks for primary keratinocyte culture. All mice were bred and maintained at the EMBL Neurobiology and Epigenetic Unit, Rome, in accordance with Italian legislation (Art. 9, 27. Jan 1992, no. 116) under license from the Italian Ministry of Health, and in compliance with the ARRIVE guidelines. Experimental protocols were approved by the EMBL Rome Ethics Committee and the Italian Ministry of Health.

### Production of recombinant SNAP and CLIP proteins

IL-31_K138A_SNAP, NGF_R121W_SNAP and the SNAP-tag were produced as previously described^14,15,41^. cDNAs for CRE recombinase or Cas9 endonuclease together with a CLIP tag were cloned into a pETM-11 vector after a SenP2 cleavage site and expressed in *E*. *Coli* as fusion protein, carrying a His6-tag-SUMO at the N-terminus. His6-SUMO-CLIP-Cre and His6-SUMO-CLIP-Cas9 constructs were recovered from cell lysate with Ni-NTA resin (Qiagen, #30210) and eluted with 250 mM imidazole. An overnight (O/N) digestion with His-tagged SenP2 protease was performed at 4 °C in order to remove the SUMO tag-protein. Excess of imidazole was removed from solution through dialysis. Digested products were incubated again with Ni-NTA resin at room temperature (RT) for 1 hr. The flow-through containing only the protein of interest was collected. Further purification was performed with Ion Exchange chromatography in a HiTrapQ HP column (GE Healthcare) and finally with size exclusion chromatography in a Superdex 75 26/60 column (GE Healthcare). The protein-containing fractions were pooled and dialysed O/N against Phosphate-buffered saline (PBS), 1 mM DTT, 5% glycerol. Dialysed proteins were then concentrated (Amicon Ultra 10 kDa, Merck-Millipore), aliquoted, snap-frozen and stored at −80 °C.

### Primary keratinocyte culture

Primary keratinocytes were isolated from adult WT C57BL/6J, Rosa26^LSL-ChR2-YFP^ and Rosa26^LSL-ChR2-YFP::IL31RA−/−^ mice as previously described^42,43^. Briefly, mice were sacrificed using CO_2_ and skins from tails were dissected and digested O/N in a solution of dispase II at 4 mg/mL (Sigma # D4693) dissolved in defined Keratinocyte-Serum Free Media (1X) (KSFM; Gibco #10744-019) containing 1% penicillin/streptomycin antibiotic and 1 mL of supplied growth supplements. The next day, the epidermis was separated from the dermis and further digested for 15 minutes at RT in a small amount of TrypLE Express solution (Gibco # 12604013), to allow gentle dissociation of primary keratinocytes from the epidermal sheet. Cells were filtered through 100 μm cell strainers (Falcon), centrifuged (4 °C, 1200 RPM, 10 minutes), and plated on μ-Dish 35 mm ibidiTreat dishes (Ibidi) or 96-well plates coated with rat-tail collagen I coating solution (Sigma # 122-20). Cells were kept in KSFM in a humidified 37 °C cell incubator with 7% CO_2_ and media was changed every day.

### *In-vitro* labelling

For keratinocyte labelling, IL-31_K138A_SNAP, NGF_R121W_SNAP or SNAP were coupled with an excess of BG-Surface^549^ at a 1:1.5 molar ratio (NEB # S9112) for 2 hours at 37 °C in PBS (pH 7.4) or CIB buffer (NaCl 140 mM; KCl 4 mM; CaCl2 2 mM; MgCl2 1 mM; NaOH 4.55 mM; Glucose 5 mM; HEPES 10 mM; pH 7.4). The coupling reaction was passed through a PD MiniTrap G-25 column (GE Healthcare #28-9180-07) to remove the excess of unbound BG. Cells from wild type mice were incubated with the coupling reaction for 2 hours at 37 °C, then washed 3 times in PBS. For N2a cell labelling, NGF_R121W_SNAP was coupled with an excess of BG-Surface^549^ at a 1:1.5 molar ratio for 2 hours at 37 °C in CIB buffer. The coupling reaction was passed through a PD MiniTrap G-25 column to remove the excess of unbound BG. N2a cells were incubated with NGF_R121W_SNAP-BG^549^ for 1–2 hours at 37 °C, then washed 3 times in PBS. 1 µg/ml Hoechst was used for nuclear staining. All images were acquired with a Leica SP5 confocal microscope. For each sample, at least 80 images (for a thickness of at least 40 µm) were acquired and then stacked together with maximum intensity using ImageJ. Positive cells were clearly distinguished from the background and were counted using the multi-point tool.

### Selective cross-linking reaction (S-CROSS)

CLIP-Cre or CLIP-Cas9 were coupled to cross-linkers at a molar ratio of 1:1.5 (CLIP-protein:linker) for 2 hours at 37 °C in PBS (pH 7.4) in the presence of 1 mM DTT. The reaction was then passed through a PD MiniTrap G-25 column to remove the excess of unbound linker and DTT, and concentrated (Amicon Ultra 10 kDa or 100 kDa, Merck-Millipore). Concentration of CLIP protein bound to the cross-linker was assessed using a NANODROP ND-8000 or by quantitative densitometry analysis of SDS-PAGE with ImageJ. The complex was then reacted with SNAP-ligands (IL-31_K138A_SNAP, NGF_R121W_SNAP or SNAP) overnight at room temperature in agitation at a 1:0.8 ratio (CLIP-protein-linker:SNAP-ligand) to minimize the presence of free ligand. Final product was analysed by SDS-PAGE. SNAP-capture magnetic beads (NEB #S9145S) were used to remove excess of free-ligand when this was present at the end of the process.

### *In-vitro* primary keratinocyte treatment with CLIP-Cre S-CROSS

Cross-linked complexes or CLIP-Cre alone were diluted *in-vitro* in keratinocytes serum free media to the desired concentration (2 μM) in a final volume of 100 μl. 36–48 hr after plating, adult primary keratinocytes were co-incubated with 100 μl of S-CROSS overnight in a humidified 37 °C cell incubator with 7% CO_2_. For TAT-Cre treatment, 1 μl (~10 unit = ~2.3 μM) of TAT-Cre (Merck-Millipore #SCR508) was diluted *in-vitro* in 100 μl of KSFM and successively co-incubated with cultured primary keratinocytes O/N in a humidified 37 °C cell incubator with 7% CO_2_. The day after, cells were washed 3 times with PBS-Heparin (1 mg/ml solution). Media was changed every day. On the fifth day after treatment, cells were imaged using a Nikon Eclipse Ti-S inverted microscope or a Leica SP5 confocal microscope. All images were analysed with ImageJ. All *in-vitro* assays were carried out in 96-well plates.

### *In-vivo* treatment with CLIP-Cre S-CROSS

Cross-linked complexes were diluted *in-vitro* in PBS to the desired concentration (5 μM; 0.85 mg/kg) in a final volume of 30 μl and subcutaneously injected into the ear of the mice. Samples were injected at the midpoint of the ear, but diffusion of the solution was observed over the whole ear after injection.

For TAT-Cre treatment, 2 μl (~20 unit = ~5.6 μM) of TAT-Cre were diluted *in-vitro* in a final volume of 45 μl of PBS and subcutaneously injected into the ear of the mice. After three weeks from the injection, the ear was dissected and the outer layer of the skin was directly whole mounted on a glass slide using 99% Glycerol (Sigma) and imaged with a Leica SP5 confocal microscope. 1 µg/ml DAPI (Invitrogen # D1306) was used to stain nuclei. All images were analysed with ImageJ.

### Cas9 *in-vitro* digestion assay

*In-vitro* digestion assay was performed as previously described^23^. Briefly, Cas9 protein or CLIP-Cas9 (30 nM) were mixed *in-vitro* with chemically synthesized Atat1 crRNA and trRNA (30 nM) and incubated with Atat1 PCR amplicons (30 ng/μl) at 37 °C for 1 hr in a Cas9 Nuclease Reaction Buffer (NEB). After nuclease reaction, mixture was treated with RNase A (5 mg/ml) and further incubated at 37 °C for 30 minutes to remove RNA. Reactions were stopped with 6× DNA loading buffer containing 30% glycerol, 1.2% SDS and 250 mM EDTA, and analyzed by electrophoresis in a 2% agarose gel.

### Electroporation of RNP complexes and DNA in primary keratinocytes

Electroporation was carried out using the AMAXA Human Keratinocyte NUCLEOFECTOR Kit (Lonza) following the manufacturer’s instructions. CLIP-Cas9 or Cas9 protein (20 μM) were incubated *in-vitro* with chemically synthesized Atat1 crRNA (13 μM) and trRNA (13 μM) in a final volume of 5 μl at 37 °C for 45 minutes in gentle agitation^44^. Primary keratinocytes were isolated as described above and prior to plating were resuspended in 100 μl of NUCLEOFECTOR solution together with CLIP-Cas9 or Cas9 ribonucleoprotein complexes. For DNA electroporation, keratinocytes were resuspended in 100 μl of NUCLEOFECTOR solution together with plasmids (2 μg - pPB-U6-Atat1) expressing the sgRNA of interest under the U6 promoter^45^ prior plating. Cells were electroporated selecting the NUCLEOFECTOR program T-24 (NUCLEOFECTOR Device I) and transferred immediately into coated μ-Dish 35 mm ibidiTreat dishes (Ibidi) or coated 12-well plates. Cells were kept in KSFM in a humidified 37 °C cell incubator with 7% CO_2_ and media was changed every day. After 72–96 hr genomic DNA was isolated to assess genome editing results.

### T7 Endonuclease I assay

Genomic DNA was isolated from treated keratinocytes. PCR amplicons containing the on-target site from the genomic locus of interest were amplified using described primers (Sigma Aldrich) and 10× DREAMTAQ Green Buffer (ThermoFisher Scientific), 0.25 mM each dNTP, 0.2 μM each primer and 0.025 U/μL DREAMTAQ DNA polymerase (ThermoFisher Scientific). The reaction was incubated in a thermal cycler programmed for 5 minutes at 95 °C followed by 34 cycles of 98 °C for 30 seconds, 60 °C for 30 seconds and 72 °C for 30 seconds then a final extension at 72 °C for five minutes. PCR fragments were separated by electrophoresis on a 2% Agarose gel, extracted from gel (QIAquick Gel Extraction Kit, Qiagen # 28115) and quantified at NANODROP ND-8000. 500 ng of each amplicon were diluted to 19 μL with 10× NEB Buffer 2 and water. The amplicon was denatured and rehybridized in a thermal cycler programmed to incubate for 10 minutes at 95 °C for 10 minutes followed by 1 minute each at 85 °C, 75 °C, 65 °C, 55 °C, 45 °C, 35 °C, and 25 °C with a 2 °C/second ramp rate. 1 μL of T7E1 (10 U/μL) (NEB #M0302S) was added and the reactions were incubated at 37 °C for 30 min. Each sample was then analyzed by electrophoresis on a 2% agarose gel, stained with Ethidium Bromide, and imaged at CHEMIDOC (Biorad).

### TIDE analysis

PCR amplicons of on-target sites were amplified and purified as previously described. Sanger traces were generated by Eurofins Genomics and analyzed with the TIDE webtool (http://tide.nki.nl)^24^. Default parameters were used for the analysis.

### Primary keratinocyte RNP internalization imaging

Primary keratinocytes were cultured as described above. 48 hr after plating, cells were incubated with 2 μM of S-CROSS complexes or CLIP-Cas9 (linker #6) (with or without Atat1 crRNA and trRNA) in a final volume of 100 μL of keratinocytes serum free media for 2 hours in a humidified 37 °C cell incubator with 7% CO_2_. After incubation, cells were washed 3 times with PBS-Heparin (1 mg/ml solution) and live imaged with a Leica SP5 confocal microscope. For protamine experiments, cross-linked RNP complexes or CLIP-Cas9 (with Atat1 crRNA and trRNA) were mixed *in-vitro* with an excess of native protamine (1:2 molar ratio) in PBS or CIB buffer (pH = 7.4) for 45 minutes at 37 °C. Reactions were diluted to 2 μM in a final volume of 100 μL of keratinocytes serum free media and applied to cells for 2 hours in a humidified 37 °C cell incubator with 7% CO_2_. After incubation cells were washed 3 times with PBS-Heparin (1 mg/ml solution) and live imaged with a Leica SP5 confocal microscope. For ppTG21 peptide, cross-linked RNP complexes or CLIP-Cas9 (with or without Atat1 crRNA and trRNA) were mixed *in-vitro* with an excess of 30 molar equivalent of ppTG21 peptide in PBS or CIB buffer (pH 7.4) for 5 min at RT. Successively, reactions were diluted to 2 μM in a final volume of 100 μL of keratinocytes serum free media and applied to cells for 2 hours in a humidified 37 °C cell incubator with 7% CO_2_. After incubation cells were washed 3 times with PBS-Heparin (1 mg/ml solution) and live imaged with a Leica SP5 confocal microscope. 1 µg/ml Hoechst was used for nuclear staining.

### *In-vitro* electroporated primary keratinocyte treatment with CLIP-Cas9 S-CROSS

CLIP-Cas9 cross-linked complexes were diluted *in-vitro* in keratinocyte serum free media to the desired concentration (2 μM) in a final volume of 100 μl. 36 hr after plasmid electroporation, adult primary keratinocytes were incubated with 100 μl of S-CROSS overnight in a humidified 37 °C cell incubator with 7% CO_2_. After incubation cells were washed 3 times with PBS-Heparin (1 mg/ml solution). After 72–96 hr, BFP positive cells were FACS sorted and genomic DNA was isolated to assess genome editing results. For ppTG21 peptide treatment, CLIP-Cas9 cross-linked complexes were mixed *in-vitro* with an excess of 30 molar equivalent of ppTG21 peptide for 5 min at RT prior incubation with cultured keratinocytes.

### AAV production

Recombinant AAV1/2 carrying U6-sgRNA sequence targeting the mouse ß-Actin gene and the monomeric Green Fluorescent Protein (mEGFP) sequence flanked by 1 kb sequences homologous to ß-Actin^29^ as a cargo was produced in HEK293 cells as described previously^46,47^. Cells were harvested 5 days post infection, lysed with Triton X-100 at 0.5%, nuclease treated, concentrated by tangential flow filtration, and purified using isopycnic ultracentrifugation^48^. Vector genome titration was performed using Q-PCR with primers targeting the promoter region of the viral cargo^46^.

### *In-vitro* N2a cell treatment with CLIP-Cas9 S-CROSS

At day 1, N2a cells were plated in a 96-well plate at 60–70% confluence and left recovering in a humidified 37 °C cell incubator with 5% CO_2_. A plasmid encoding for TrkA receptor was transfected 8–10 hr after plating using Lipofectamine 2000 Transfection Reagent (ThermoFisher Scientific). 24 hr after plating, cells were infected with 1 uL of AAV1/2::HDR in 30 uL of PBS and returned to the incubator. 30 minutes after infection, 70 uL of Dulbecco’s Modified Eagle Medium (DMEM, Gibco) supplemented with 10% Fetal Bovine Serum (FBS) and 1% Penicillin/Streptomycin (P/S) were added. After 8–24 hr, cells were starved for 30 min in serum free media (DMEM). NGF^SNAP::CLIP^Cas9 cross-linked complexes (or CLIP-Cas9) were diluted *in-vitro* in CIB buffer to the desired concentration (1–2 μM) in a final volume of 30 μl and immediately applied to starved N2a cells. For ppTG21 peptide co-treatment, NGF^SNAP::CLIP^Cas9 cross-linked complexes were mixed *in-vitro* with an excess of 30 molar equivalent of ppTG21 peptide in CIB buffer (pH 7.4) for 5 min at RT prior treatment. 30–60 minutes after treatment, 70 uL of DMEM supplemented with 10% FBS and 1% P/S were added. Cells were left in a humidified 37 °C cell incubator with 5% CO_2_ for 2–4 days (48–96 hr) and live imaged with a Leica SP5 confocal microscope.

### *In-vivo* treatment with CLIP-Cas9 S-CROSS

Cross-linked complexes were diluted *in-vitro* in CIB buffer to the desired concentration (5 μM) together with 5 uL of AAV1/2::HDR in a final volume of 10–15 μl and subcutaneously injected into the ear of the mice. For dual AAV treatment, 5 μl of AAV1/2::HDR were mixed with 5 uL of AAV1/2::Cas9 and subcutaneously injected into the ear of the mice. After 15–21 days from the injection, the ear was dissected and the outer layer of the skin was fixed for 30 minutes in PFA 4%. After fixation, the sample was washed 3 times with PBS and cleared overnight in a ScaleA2 solution (4 M urea, 10% (wt/vol) glycerol and 0.1% (wt/vol) Triton X-100; pH = 7.7)^49^. The following day the samples were mounted on a glass slide and imaged with a Leica SP5 confocal microscope. 1 µg/ml DAPI (Invitrogen # D1306) was used to stain nuclei. All images were analysed with ImageJ.

### SDS-PAGE and western blotting

To assess the coupling reaction, CLIP-Cre or CLIP-Cas9 were coupled with an excess of BC-Surface^488^ or BC^TMR^ at a 1:1.5 molar ratio (NEB #S9232S; #S9219S) for 1 hour at 37 °C in PBS (pH 7.4). The coupling reactions were analyzed by SDS-PAGE on a precast acrylamide gel (BioRad #456-9034), along with known concentrations of proteins alone. The bands corresponding to the binding were visualized by gel fluorescence. To assess the cross-linking reaction, cross-linked samples were analyzed by SDS-PAGE on a precast acrylamide gel, alongside a sample of known concentration. Quantification was performed as previously described. All the samples were visualized by Coomassie staining.

For SNAP-ligands and cross-linked complexes internalization, keratinocytes were treated for 2 hours at 37 °C, washed three times with PBS-Heparin (1 mg/mL) and then collected by scraping in 1 mL of cold PBS. The cell suspension was centrifuged and lysed in RIPA Buffer (Sigma, #R0278) with proteases inhibitor cocktail (Roche #11873580001). For all the experiments, the total lysate was separated on 10% or 8% SDS-PAGE gel and transferred to a nitrocellulose membrane (Protran #10600007). Membranes were incubated with anti-SNAP antibody (NEB #P9310S) or with anti-ß-actin antibody (Cell Signaling #4970). Bands were visualized using the ECL detection system (Amersham #RPN2106); band density was calculated using ImageJ.

### Flow cytometry

To confirm gene editing at the Atat1 locus, BFP positive primary keratinocytes were isolated for genomic DNA isolation using a BD FACSAria III cell sorter (Becton Dickinson, San Jose, USA). The cells were incubated with EDTA 0.05% solution (in DPBS) for 10–15 minutes at 37 °C and then incubated with 0.05% trypsin, 0.02% EDTA until detachment. After trypsinization, cells were suspended in FACS buffer (1x PBS; 10% FBS; 2 mM EDTA) and sorted directly into lysis buffer for DNA extraction with a 100 μm nozzle at 20 PSI and sorting mask values of yield = 0, purity = 32, and phase = 0. Physical parameters were measured by FSC and SSC from a 488 nm 100 mW laser and BFP was excited by a 405 nm 100 mW laser and detected through a 450/50 band-pass filter. To select the BFP expressing population to be sorted, the following gating scheme was used: an initial viability gate of SSC-A vs. FSC-A, then down selection by 2 coincidence gates of FSC-W vs FSC-H and SSC-W vs SSC-H, followed by a BFP positive inclusive gate from an SSC-A vs BFP dot plot. Untreated primary keratinocytes were used to determine the non-BFP keratinocytes so that BFP positive cells could be gated.

## Supplementary information

Supplementary Information
