# Supplementary material for: A ligand-based system for receptor-specific delivery of proteins
Source: Sci Rep. 2019 Dec 16;9:19214. doi: 10.1038/s41598-019-55797-1 (PMC6915567; doi:10.1038/s41598-019-55797-1)
Supplement: Supplementary file 1 — Supplementary Information [file 41598_2019_55797_MOESM1_ESM.pdf]

A ligand-based system for receptor-specific delivery of proteins

Mariano Maffei[1*+,] Chiara Morelli[1,6+], Ellie Graham[1], Stefano Patriarca[1], Laura Donzelli[1], Balint Doleschall[1], Fernanda de Castro Reis[1], Linda Nocchi[1], Cora H. Chadick[1], Luc Reymond[2,3], Ivan R. Corrêa Jr.[4], Kai Johnsson[5], Jamie A. Hackett[1] & Paul A. Heppenstall[1*]

Supplementary Information

Methods

Fig. S1. Proteins labelling and cross-linking.

Fig. S2. CLIP-Cre injection in reporter mice.

Fig. S3. In-cell Cas9 RNP delivery and cross-linking.

Fig. S4. CLIP-Cas9 internalization and gene-editing.

Fig. S5. FACS and in-cell gene editing.

Fig. S6. NGF binding to N2a cells and CLIP-Cas9 treatment of N2a cells.

Table S1. Cross-linkers.

Methods

Synthesis of linkers

Synthesis of linker #1 (BG-BC)[19], linker #4 (BG-647-BC)[50], linker #3 (BG-PEG-(S-S)-Biotin-PEG-BC)[20] and linker #5 (BG-PEG-Biotin-PEG-BC)[20] was previously described.

Linker #2 (BG-PEG-649-PEG-BC) was prepared by reacting BG-PEG-BC[51] with commercially available DY-649 NHS (Dyomics GmbH, Jena, Germany). Briefly, BG-PEG-BC (1.7 mg, 1.6 μmol) was dissolved in anhydrous DMF (1.0 mL). DY-649 NHS (2.6 mg, 2.6 μmol) was added followed by triethylamine (0.6 μL, 4.8 μmol). The reaction mixture was stirred overnight at room temperature. The solvent was removed under vacuum and the

products purified by reversed-phase HPLC on a VYDAC 218TP series C18 column (22 x 250 mm, 10 µm particle size) at a flow rate of 25 mL/min using a 0.1 M aqueous TEAB buffer/acetonitrile gradient (5 to 95% acetonitrile over 25 min). Yield: BG-PEG-649-PEG-BC (57%), ESI-MS m/z 1872.6695 (calc. for $C83H111N17O25S4^-$, m/z 1872.6747). High-resolution mass spectrum was recorded by electrospray ionization (ESI) on a Thermo Scientific™ Q Exactive™ Plus Hybrid Quadrupole-Orbitrap™ Mass Spectrometer.

Linker #6 (BG-TMR-PEG-BC) was prepared as following: all chemical reagents and solvents for synthesis were purchased from commercial suppliers (Sigma-Aldrich, Fluka, Acros) and were used without further purification or distillation. The composition of mixed solvents is given by the volume ratio (v/v). Thin layer chromatography (TLC) was performed on TLC-aluminum sheets (Silica gel 60 F254). 1H nuclear magnetic resonance (NMR) spectra were recorded on a Bruker DPX 400 (400 MHz for 1H) with chemical shifts (δ) reported in ppm relative to the solvent residual signals of DMSO-d6 (2.50 ppm for 1H), and coupling constants reported in Hz. High resolution mass spectra (HRMS) were measured on a Micromass Q-TOf Ultima spectrometer with electron spray ionization (ESI). Liquid chromatography coupled to mass spectrometry (LC-MS) was performed on a Shimadzu MS2020 connected to a Nexera UHPLC system equipped with a Waters ACQUITY UPLC BEH C18 (1.7 µm, 2.1 x 50 mm) column or a Waters ACQUITY UPLC BEH PHENYL (1.7 µm, 2.1 x 50 mm). Buffer A: 0.05% HCOOH in H2O Buffer B: 0.05% HCOOH in ACN. Analytical gradient was from 10% to 90% B within 5 min with 0.6 mL/min flow unless otherwise stated. Preparative RP-HPLC was performed on a Dionex system equipped with an UVD 170U UV-Vis detector for product visualization on a Waters Symmetry C18 (5 µm, 3.9 x 150 mm) Column. Buffer A: 0.1% w/v TFA in H2O Buffer B: acetonitrile. Typical gradient was from 10% to 90% B within 30 min with 3 ml/min flow.

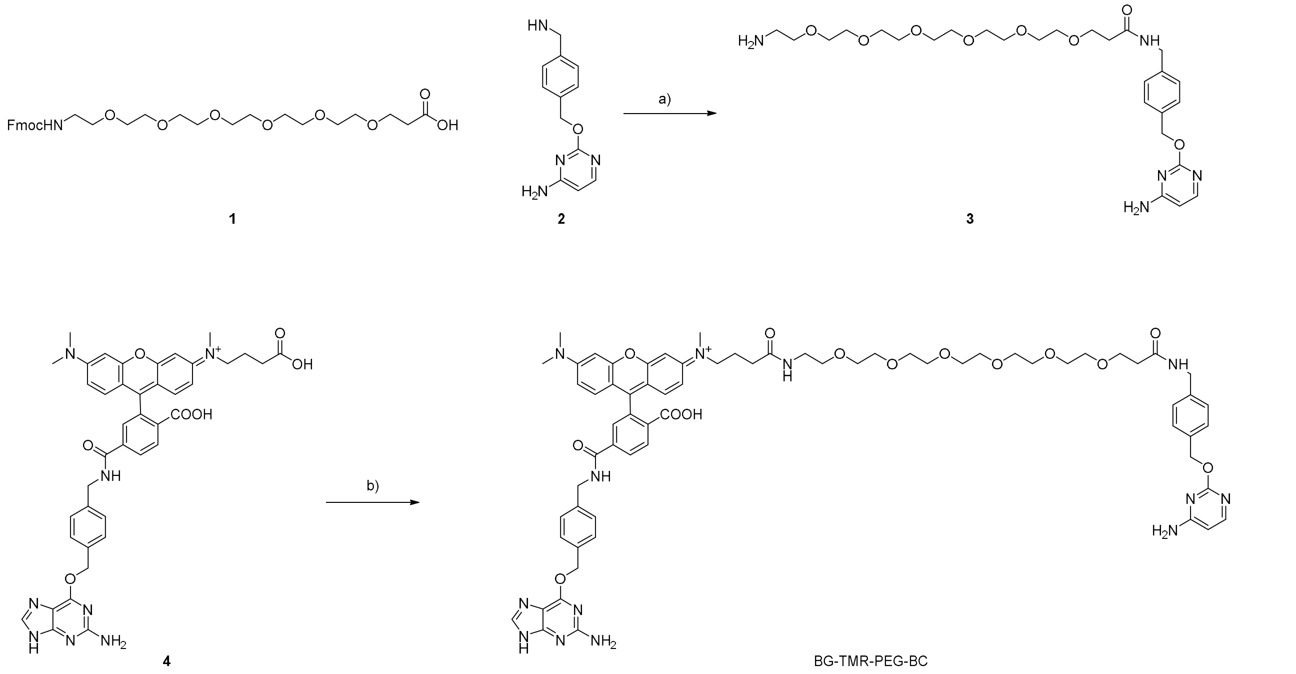

Scheme 1. Synthesis of linker #6

a) i. HBTU, DIPEA, DCM; ii) DBU, MeCN. b) TSTU, DIPEA, 3, DMSO

3 A solution of Fmoc-PEG-COOH 1 (29 mg, 1.0 eq.), BC-NH2 2 (14 mg, 1.2 eq), DIPA (35 ul, 4 eq.) and HBTU (25 mg, 1.2 eq.) in DCM (1 ml) was stirred for 30 min. The reaction was diluted with 20 ml DCM and extracted with water (20 ml). The organic phase was dried and evaporated. The residue was suspended in MeCN (1 ml) and treated with DBU (15 ul, 2.0 eq.) after 5 minutes, AcOH (15 ul) was added. The product 3 was purified by HPLC and lyophilized. Yield 16 mg (as TFA salt, 47%). 1H NMR (400 MHz, DMSO-d6) δ 8.66 (s, 2H), 8.40 (t, J = 6.0 Hz, 1H), 7.98 (d, J = 6.9 Hz, 1H), 7.81 (s, 3H), 7.49 – 7.38 (m, 2H), 7.34 – 7.23 (m, 2H), 6.35 (d, J = 6.9 Hz, 1H), 5.42 (s, 2H), 4.28 (d, J = 5.9 Hz, 2H), 3.67 – 3.45 (m, 24H), 2.98 (q, J = 5.5 Hz, 2H), 2.39 (t, J = 6.4 Hz, 2H). MS (ESI) calculated for C27H44N5O8 [M+H]+ 566.3; found 566.3.

BG-TMR-PEG-BC

4 (1.5 mg, 1.0 eq.)[25] (U.S. Patent Application No. 15/550,239) was dissolved in 150 ul DMSO, treated with DIPEA (0.3 ul, 2.0 eq.) and TSTU (0.1 mg, 1.5 eq). After 5 min, 3 (1.4 mg, 1.1 eq) was added and the reaction was shaken for an extra 30 min. The product was purified by HPLC and lyophilized. Yield: 1.2 mg (48%). HRMS (ESI) calculated for C68H80N13O14 [M+] 1302.5942; found 1302.5979.

Oligos and single guides RNA

Oligos used for PCR amplification of mouse Atat1 locus were:

5’- CTA TTG TAG ATG AGC TGG GC -3’ (Atat1 Forward primer)

5’- CTA TTG TAG ATG AGC TGG GC -3’ (Atat1 Reverse primer)

Single guide RNA targeting mouse Atat1: 5’- tcagtatgtaaataacgtgc – 3’.

Single guide RNA targeting mouse ß-actin: 5’- tggcgaactatcaagacaca – 3’.

Donor Template for ß-actin-mEGFP:

tgcagctccttcgttgccggtccacacccgccaccaggtaagcagggacgccgggcccagcgggccttcgctctctcgtggct
agtacctcactgcagggtcctgaggatcactcagaacggacaccatgggcgggtggagggtggtgccgggccgcggagc
ggacactggcacagccaactttacgcctagcgtgtagactctttgcagccacattcccgcggtgtagacactcgtgggcccgc
tcccgctcggtgcgtggggcttggggacacactagggtcgcggtgtgggcatttgatgagccggtgcggcttgcgggtgttaa
aagccgtattaggtccatcttgagagtacacagtattgggaaccagacgctacgatcacgcctcaatggcctctgggtctttgtc
caaaccggtttgcctattcggcttgccgggcgggcgggcgggcgggcgggcgcggcagggccggctcggccgggtgggg
gctgggatgccactgcgcgtgcgctctctatcactgggcatcgaggcgcgtgtgcgctagggagggagctcttcctctcccct
cttcctagttagctgcgcgtgcgtattgaggctgggagcgcggctgcccggggttgggcgagggcggggccgttgtccggaa
ggggcggggtcacagtggcacgggcgccttgtttgcgcttcctgctgggtgtggtcgcctcccgcgcgcgcacaagccgccc
gtcggcgcagtgtaggcggagcttgcgcccgtttggggagggggcggaggtctggcttcctgccctaggtccgcctccgggc
cagcgtttgccttttatggtaataatgcggccggtctgcgcttcctttgtcccctgagcttgggcgcgcgcccccctggcggctcgag
cccgcggcttgccggaagtgggcagggcggcagcggctgctcttggcggccccgaggtgactatagccttcttttgtgtcttgat
agttcgccatgcatGTGAGCAAGGGCGAGGAGCTCTTCACCGGGGTGGTGCCCATCCTGGT

CGAGCTGGACGGCGACGTAAACGGCCACAAGTTCAGCGTGTCCGGCGAGGGCGAG
GGCGATGCCACCTACGGCAAGCTGACCCTGAAGTTCATCTGCACCACCGGCAAGCT
GCCCGTGCCCTGGCCCACCCTCGTGACCACCCTGACCTACGGCGTGCAGTGCTTCA
GCCGCTACCCCGACCACATGAAGCAGCACGACTTCTTCAAGTCCGCCATGCCCGAA
GGCTACGTCCAGGAGCGCACCATCTTCTTCAAGGACGACGGCAACTACAAGACCCG
CGCCGAGGTGAAGTTCGAGGGCGACACCCTGGTGAACCGCATCGAGCTGAAGGGCA
TCGACTTCAAGGAGGACGGCAACATCCTGGGGCACAAGCTGGAGTACAACTACAACA
GCCACAACGTCTATATCATGGCCGACAAGCAGAAGAACGGCATCAAGGTGAACTTCA
AGATCCGCCACAACATCGAGGACGGCAGCGTGCAGCTCGCCGACCACTACCAGCAG
AACACCCCCATCGGCGACGGCCCCGTGCTGCTGCCCGACAACCACTACCTGAGCAC
CCAGTCCAAGCTGAGCAAAGACCCCAACGAGAAGCGCGATCACATGGTCCTGCTGG
AGTTCGTGACCGCCGCCGGGATCACTCTCGGCATGGACGAGCTGTACAAGgatgacgat
atcgctgcgctggtcgtcgacaacggctccggcatgtgcaaagccggcttcgcgggcgacgatgctccccgggctgtattccc
ctccatcgtgggccgccctaggcaccaggtaagtgacctgttactttgggagtggcaagcctggggttttcttggggatcgatgc
cggtgctaagaaggctgttcccttccacagggtgtgatggtgggaatgggtcagaaggactcctatgtgggtgacgaggccc
agagcaagagaggtatcctgaccctgaagtaccccattgaacatggcattgttaccaactgggacgacatggagaagatct
ggcaccacaccttctacaatgagctgcgtgtggcccctgaggagcaccctgtgctgctcaccgaggccccccctgaaccctaa
ggccaaccgtgaaaagatgacccaggtcagtatcccgggtaacccttctctttggccagcttctcagccacgccctttctcaatt
gtctttcttctgccgttctcccataggactcccttctatgagctgagtctcccttggatctttgcagtttctgctctttcccagacgaggtc
tttttttctctcaattgcctttctgactaggtgtttaaaccctacagtgctgtgggtttaggtactaacaatggctcgtgtgacaaagct
aatgaggctggtgataagtggccttggagtgtgtattgagtagatgcacagtaggtctaagtggagcccctgtcctgagactcc
cagcacactgaacttagctgtgttcttgcactccttgcatgtctcagatctatccatacagtttcacctgccctgagtgtttcttgtggc
tttctgaacttgacaacattatttatttttctctacagatcatgtttgagaccttcaacaccccagccatgtacgtagccatccaggct
gtgctgtccctgtatgcctctggtcgtaccacaggcattgtgatggac

Figures and Tables

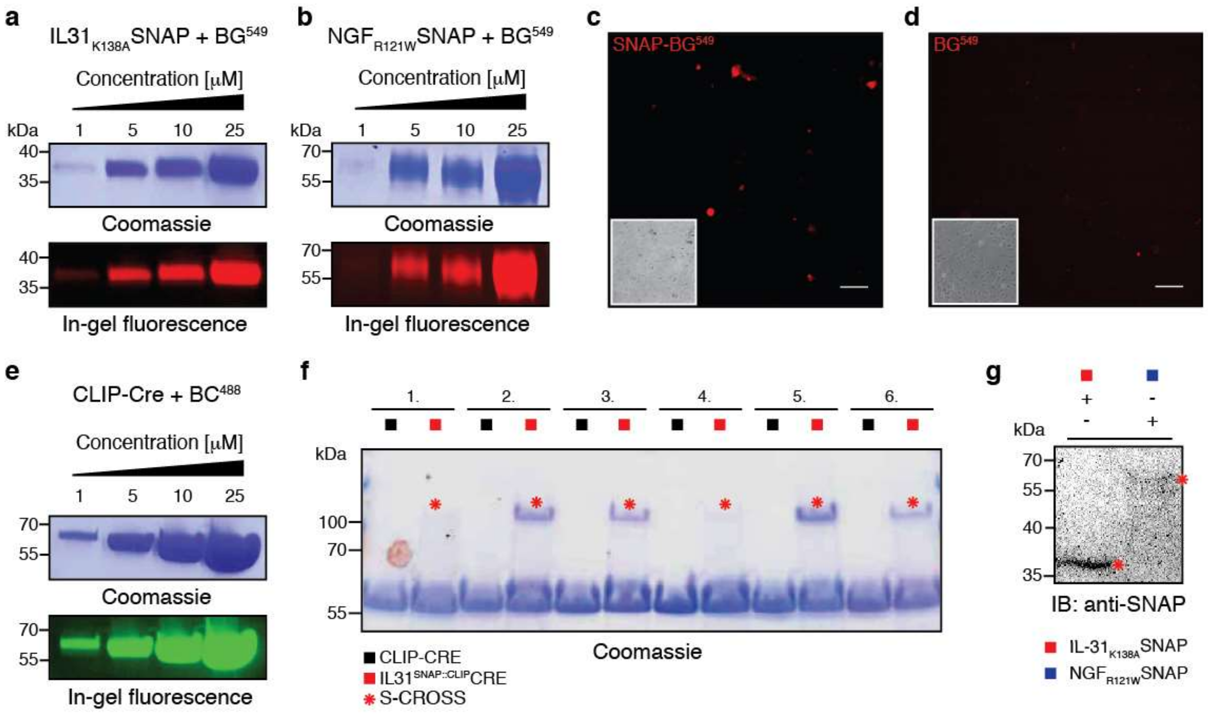

Fig. S1. IL31K138ASNAP (A) and NGFR121WSNAP (B) binding to a BG^{549} fluorophore at an increasing range of concentrations. (C) Labelled SNAP-BG^{549} binding to primary keratinocytes. The inset represents the corresponding brightfield image. Scale bars, 20 μm. (D) BG^{549} incubation with primary keratinocytes. The inset represents the corresponding brightfield image. Scale bars, 20 μm. (E) Representative Coomassie and fluorescence gel showing CLIP-Cre binding to a BC^{488} fluorophore at an increasing range of concentrations. (F) Coomassie gel showing S-CROSS of IL31K138ASNAP and CLIP-Cre (red squares) and CLIP-Cre alone (pink squares). Red asterisks indicate cross-linking. Condition 1: linker #1 BG-BC; Condition 2: linker #2 BG-PEG-649-PEG-BC; Condition 3: linker #3 BG-PEG-(S-S)-Biotin-PEG-BC; Condition 4: linker #4 BG-647-BC; Condition 5: linker #5 BG-PEG-Biotin-PEGBC; Condition 6: linker #6 BG-TMR-PEG-BC (Table S1). (G) Western blot showing IL31K138ASNAP (lane #1, red square) and NGFR121WSNAP (lane #2, blue square) internalization in primary keratinocytes after 2 hours incubation with 1 μM of each ligand.

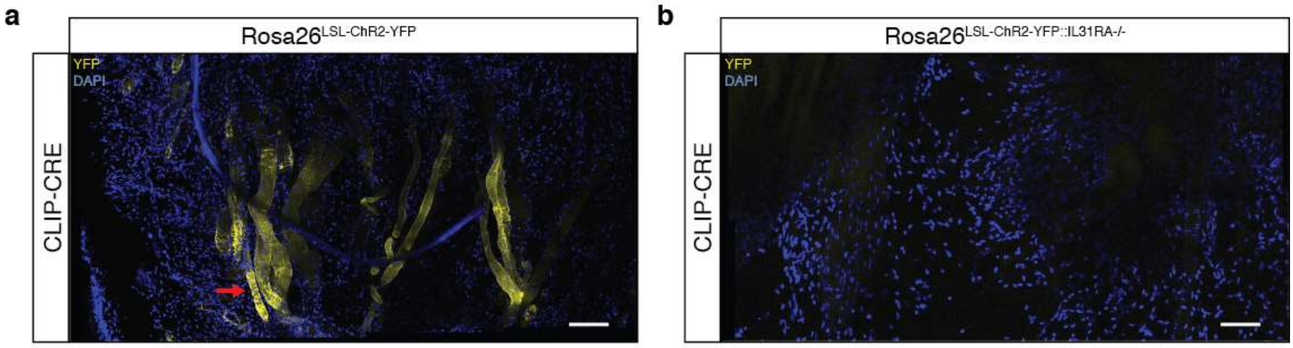

Fig. S2. YFP expression from Rosa26$^{LSL\text{-}ChR2\text{-}YFP}$ mice (A) and from double transgenic Rosa26$^{LSL\text{-}ChR2\text{-}YFP::IL31RA\text{-}/\text{-}}$ mice (B) 3 weeks after subcutaneous injection with 5 µM of CLIP-Cre. The nuclei were stained with DAPI. Scale bars, 40 µm. Red arrow indicates non-selective YFP expression.

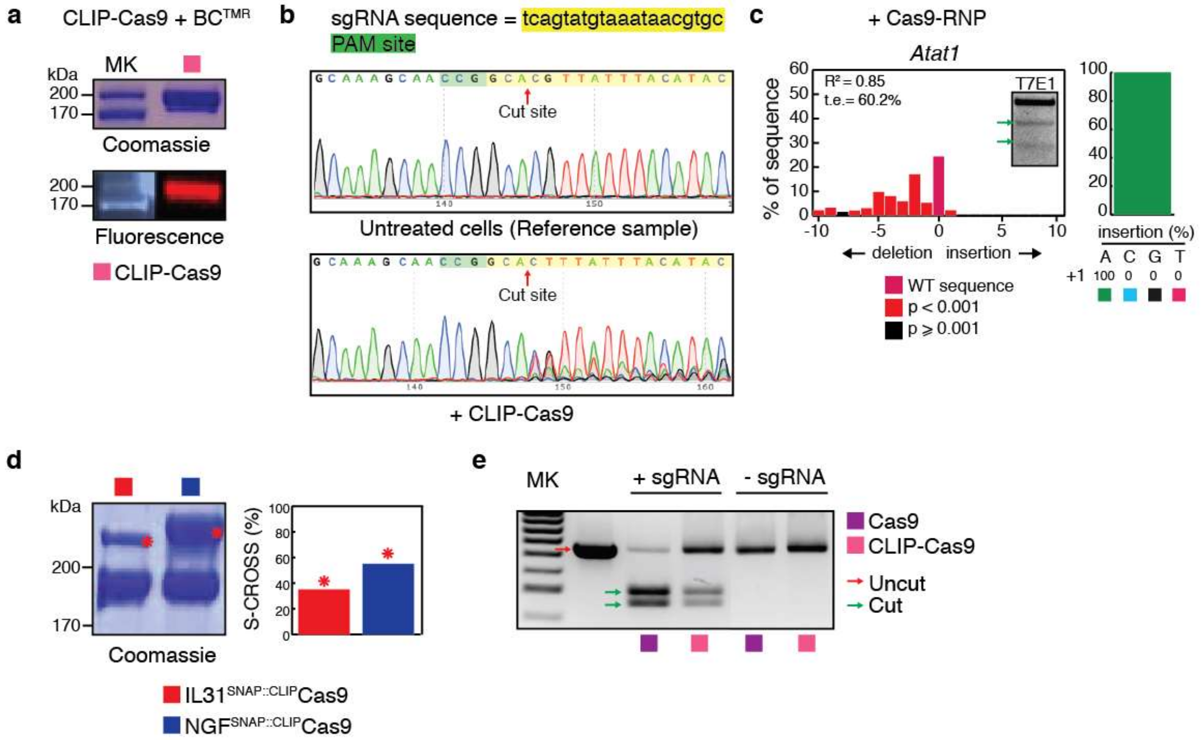

Fig. S3. (A) Representative Coomassie and fluorescence gel showing CLIP-Cas9 binding to a BC^{TMR} fluorophore. Lane #1 protein ladder. (B) Sequencing chromatograms showing the targeted Atat1 locus. Control sample (upper chromatogram) and treated sample (+ CLIP-Cas9; lower chromatogram). (C) Indel spectrum determined by TIDE of primary keratinocytes electroporated with Cas9::sgRNA targeting the *Atat1* gene. The inset show T7 endonuclease 1 assay performed on genomic DNA from electroporated keratinocytes. t.e.= total efficiency. The estimated composition of the inserted base for the +1 insertion is also shown. (D) Representative Coomassie gel and quantification (% S-CROSS) showing cross-linking complexes (red asterisks). First lane (#1) is IL-31$^{SNAP::CLIP}$Cas9, second lane (#2) is NGF$^{SNAP::CLIP}$Cas9. (E) In-vitro digestion assay of the PCR products amplified from the Atat1 locus and incubated with: Cas9::sgRNA (lane #2), CLIP-Cas9::sgRNA (lane #3), Cas9 (lane #4), CLIP-Cas9 (lane #5). Atat1 undigested PCR product is shown in lane #1.

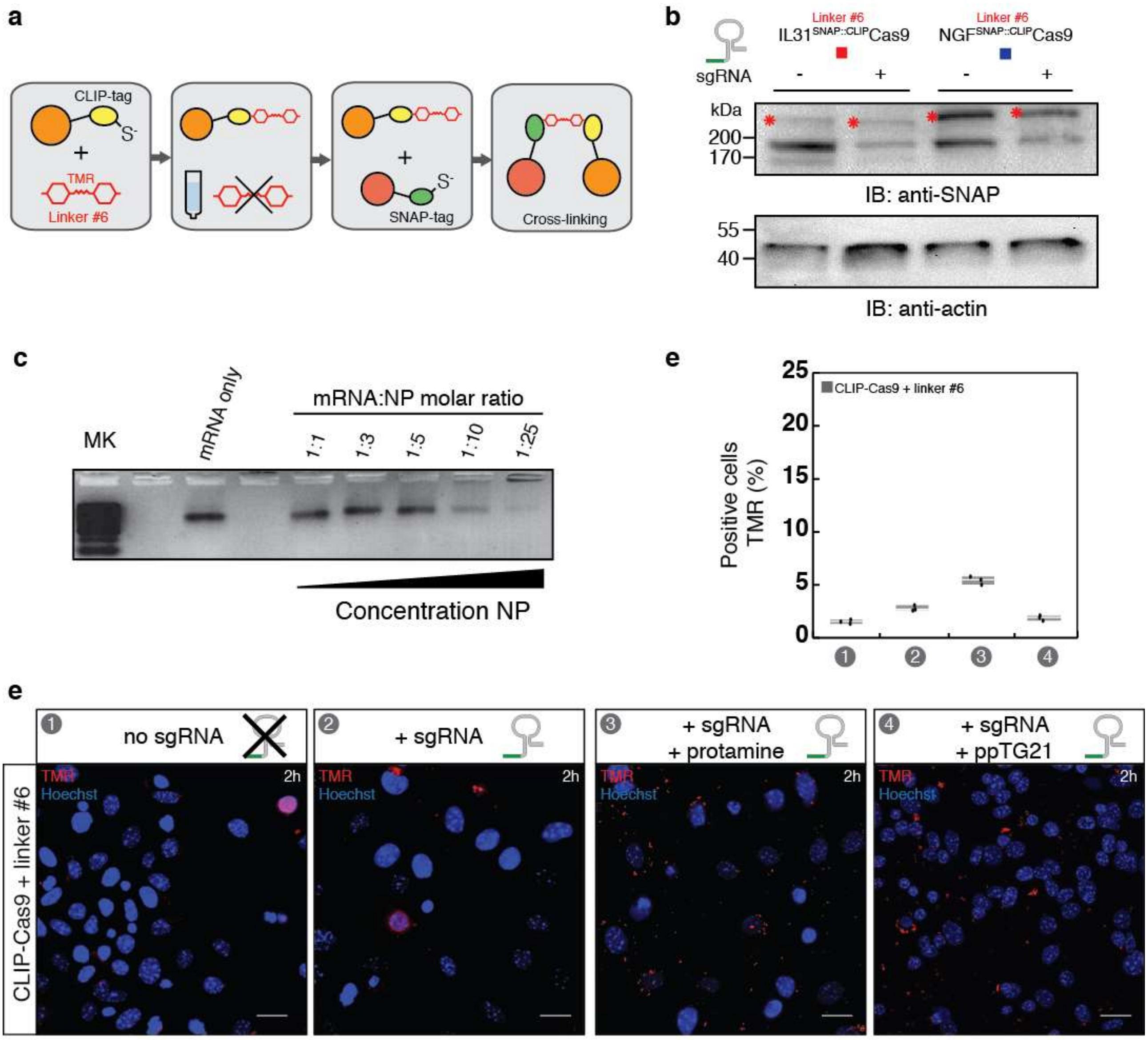

Sample	linker	peptide	indels (%)
IL31SNAP::CLIPCas9::sgRNA	○ linker #6	N/A	2.0%
IL31SNAP::CLIPCas9::sgRNA	○ linker #6	protamine	0.9%
IL31SNAP::CLIPCas9::sgRNA	○ linker #6	ppTG21	1.6%
NGFSNAP::CLIPCas9::sgRNA	○ linker #6	N/A	2.2%
NGFSNAP::CLIPCas9::sgRNA	○ linker #6	protamine	0.3%
NGFSNAP::CLIPCas9::sgRNA	○ linker #6	ppTG21	1.2%

Fig. S4. (A) Schematic representation of S-CROSS using linker #6. (B) Western blots showing IL-31$^{SNAP::CLIP}$Cas9 and NGF$^{SNAP::CLIP}$Cas9 internalization in primary keratinocytes in absence (lanes #1 and #3) and in presence (lanes #2 and #4) of Atat1 dual sgRNA after 2 hours incubation with 2 μM of each ligand. (C) RNA gel shift assay in presence of increasing molar ratio of native protamine (NP) (mRNA:NP). (D) Quantification of (E) TMR

positive cells upon 2 hour treatment with 2 μM of CLIP-Cas9 (#1 no sgRNA; #2 with sgRNA; #3 with sgRNA + Protamine; #4 with sgRNA + ppTG21). The nuclei were stained with Hoechst. Scale bars, 20 μm. The axis scale shows values from 0% to 25%. (F) Percentage of indels measured from genomic DNA sequencing.

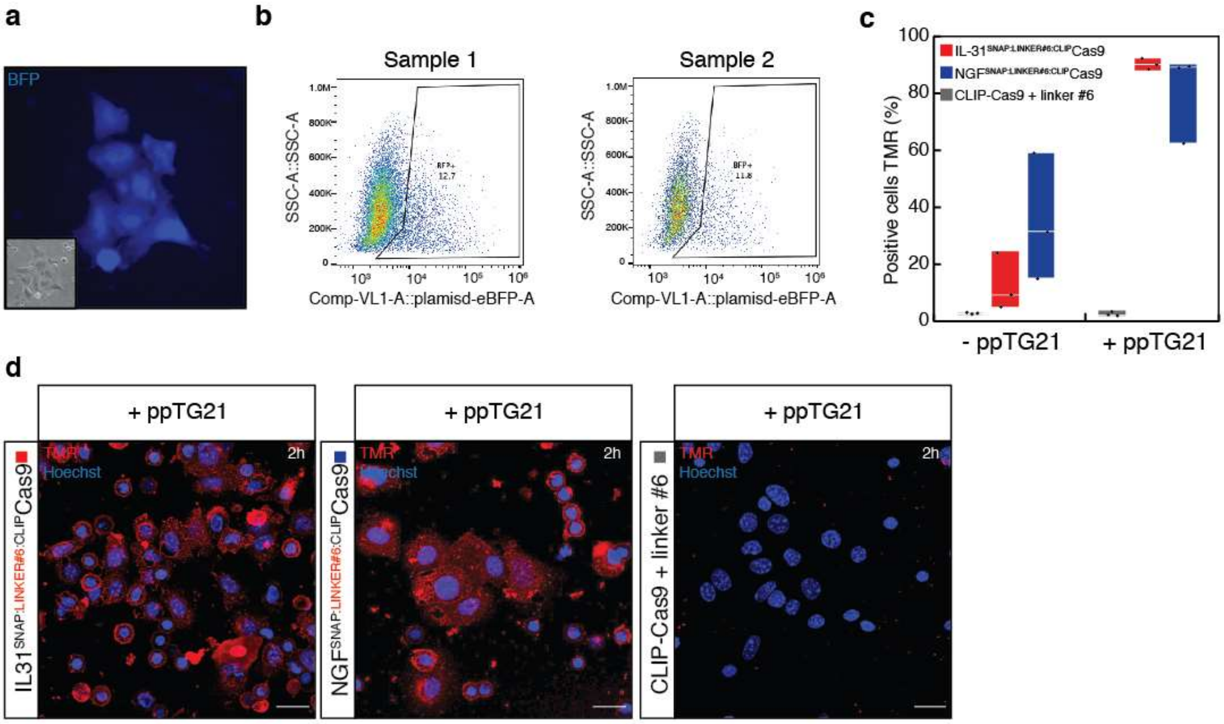

e

Sample	linker	peptide	indels (%)	av. indels (%)
IL31$^{SNAP::CLIP}$Cas9	△ linker #5	N/A	5.2%	6.2%
IL31$^{SNAP::CLIP}$Cas9	□ linker #3	N/A	6.8%	
IL31$^{SNAP::CLIP}$Cas9	○ linker #6	N/A	6.7%	
IL31$^{SNAP::CLIP}$Cas9	△ linker #5	ppTG21	9.6%	6.2%
IL31$^{SNAP::CLIP}$Cas9	□ linker #3	ppTG21	2.7%	
IL31$^{SNAP::CLIP}$Cas9	○ linker #6	ppTG21	6.4%	
NGF$^{SNAP::CLIP}$Cas9	△ linker #5	N/A	6.1%	6.6%
NGF$^{SNAP::CLIP}$Cas9	□ linker #3	N/A	11.1%	
NGF$^{SNAP::CLIP}$Cas9	○ linker #6	N/A	2.6%	
NGF$^{SNAP::CLIP}$Cas9	△ linker #5	ppTG21	19.8%	11.8%
NGF$^{SNAP::CLIP}$Cas9	□ linker #3	ppTG21	9.2%	
NGF$^{SNAP::CLIP}$Cas9	○ linker #6	ppTG21	6.5%	
only sgRNA (BFP ctrl)	N/A	N/A	3.9%	3.7%
only sgRNA (BFP ctrl)	N/A	N/A	4.0%	
only sgRNA (BFP ctrl)	N/A	N/A	3.3%	
Untreated control	N/A	N/A	1.6%	1.9%
Untreated control	N/A	N/A	2.3%	
Untreated control	N/A	N/A	1.9%	

Fig. S5. (A) Representative image of BFP expressing cells. The inset shows the respective brightfield image. (B) BFP positive cells indicated by Flow Cytometry analysis. (C) Quantification of (D) TMR positive cells upon 2 hour treatment with 2 μM of IL-31$^{SNAP::CLIP}$Cas9 (linker #6; left frame, red square), NGF$^{SNAP::CLIP}$Cas9 (linker #6; middle frame, blue square) and CLIP-Cas9 (linker #6; right frame, grey square) in presence of 30 molar excess of ppTG21 peptide. The nuclei were stained with Hoechst. Scale bars, 20 μm. (E) Percentage of indels measured from genomic DNA sequencing.

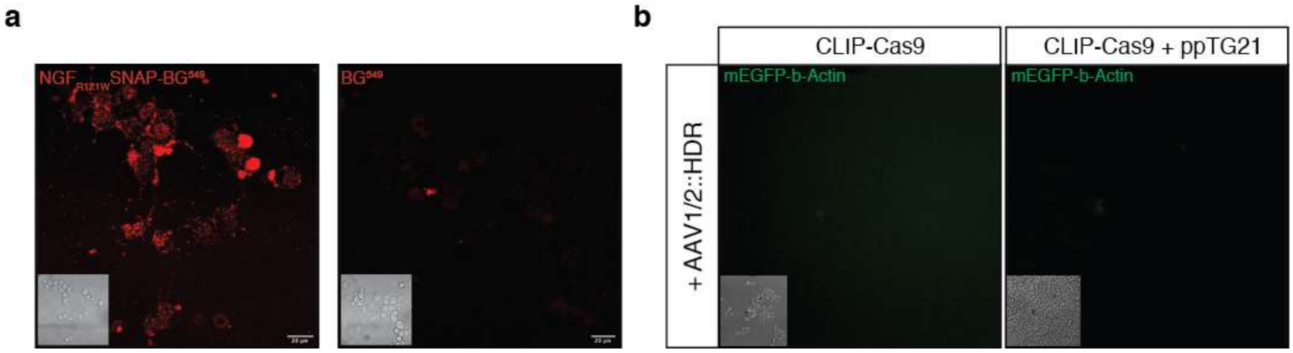

Fig. S6. (A) N2a cells over expressing NGF receptors and labelled with NGF_{R121W}SNAP-BG^{549} or BG^{549} alone. The insets represent the corresponding brightfield image. Scale bars, 20 µm. (B) Representative images of AAV1/2::HDR transduced N2a cells treated only with CLIP-Cas9 (left frame) or in the presence of ppTG21 peptide (right frame). The insets represent corresponding brightfield images.

	Linker synonym	Linker Structure	Distance (Å)
1.	BG-BC	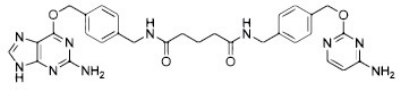	< 25
2.	BG-PEG-649-PEG-BC	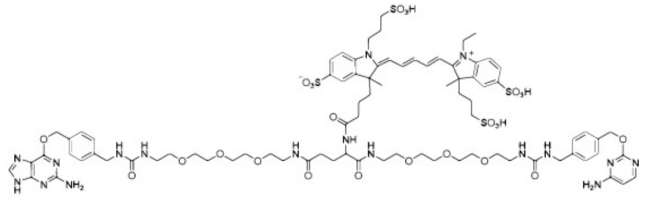	> 25
3.	BG-PEG-(S-S)-Biotin-PEG-BC	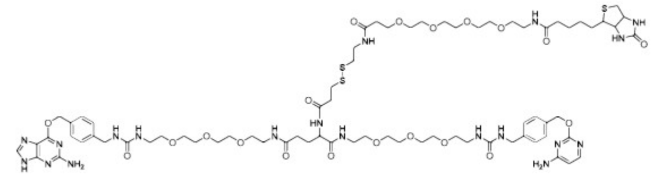	> 25
4.	BG-647-BC	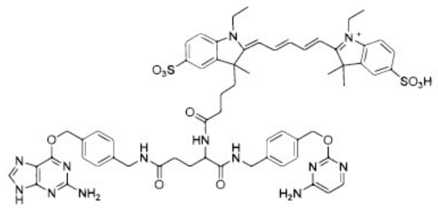	< 25
5.	BG-PEG-Biotin-PEG-BC	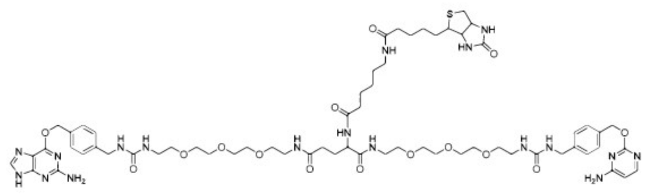	> 25
6.	BG-TMR-PEG-BC	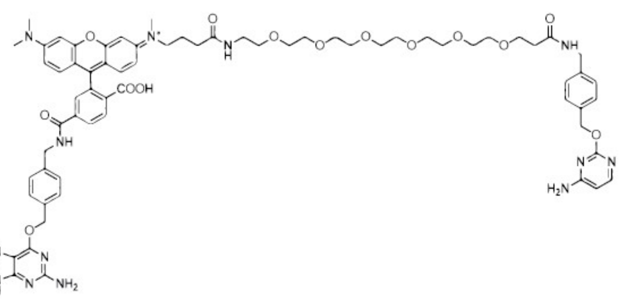	> 25

Table S1. Table showing the cross-linker synthetic probes tested for S-CROSS reactions.

References

50 Haruki, H., Gonzalez, M. R. & Johnsson, K. Exploiting ligand-protein conjugates to monitor ligand-receptor interactions. *PLoS One* **7**, e37598, doi:10.1371/journal.pone.0037598 (2012).

51 Sun, X. *et al.* Probing homodimer formation of epidermal growth factor receptor by selective crosslinking. *Eur J Med Chem* **88**, 34-41, doi:10.1016/j.ejmech.2014.07.041 (2014).
